# The role of replication-induced chromosomal copy numbers in spatio-temporal gene regulation and evolutionary chromosome plasticity

**DOI:** 10.3389/fmicb.2023.1119878

**Published:** 2023-04-20

**Authors:** Marc Teufel, Werner Henkel, Patrick Sobetzko

**Affiliations:** ^1^Synthetic Microbiology Center Marburg (SYNMIKRO), Philipps Universität Marburg, Marburg, Germany; ^2^Transmission Systems Group, Jacobs University Bremen, Bremen, Germany; ^3^DynAMic Department, Universitè de Lorraine, INRAE, Nancy, France

**Keywords:** genome editing, gene regulation, replication, chromosome architecture, transcriptomics (RNA-seq), genome rearrangement, inversion

## Abstract

For a coherent response to environmental changes, bacterial evolution has formed a complex transcriptional regulatory system comprising classical DNA binding proteins sigma factors and modulation of DNA topology. In this study, we investigate replication-induced gene copy numbers - a regulatory concept that is unlike the others not based on modulation of promoter activity but on replication dynamics. We show that a large fraction of genes are predominantly affected by transient copy numbers and identify cellular functions and central pathways governed by this mechanism in *Escherichia coli*. Furthermore, we show quantitatively that the previously observed spatio-temporal expression pattern between different growth phases mainly emerges from transient chromosomal copy numbers. We extend the analysis to the plant pathogen *Dickeya dadantii* and the biotechnologically relevant organism *Vibrio natriegens*. The analysis reveals a connection between growth phase dependent gene expression and evolutionary gene migration in these species. A further extension to the bacterial kingdom indicates that chromosome evolution is governed by growth rate related transient copy numbers.

## Introduction

Bacteria interact dynamically with the environment and adapt to external and internal conditions. The first level of adaption is the regulation of gene expression to integrate various signals in a concerted manner. One of the major regulatory mechanism is DNA topology. Here, the 3D structure of the DNA and tension within the DNA molecule is converted in more or less favorable conditions for RNAP and regulator binding. The main actors are the antagonists DNA Gyrase and Topoisomerase I (Menzel and Gellert, 1983). These enzymes remove or add helical turns to the DNA and thereby modulate tension in the DNA molecule. Activity and abundance of the antagonists are tightly regulated and change upon transition between growth phases (Balke and Gralla, 1987). Through modulation of activity and abundance of DNA Gyrase and Topoisomerase I, DNA supercoiling levels are controlled realizing a global regulation. Moreover, DNA topology can be altered locally by transcription activity in the neighborhood of promoters following the Liu Wang Model (Liu and Wang, 1987; Riebet and Raibaud, 1991; Chen and Lilley, 1999). Consequently, orientation and activity of neighboring genes and sensitivity of the affected promoter form another layer of locally organized regulation (Sobetzko, 2016; El Houdaigui et al., 2019). Transmission of regulatory information via DNA topology is locally restricted due to its nature. Longer distances requiring no direct contact of DNA molecules are spanned by DNA binding proteins comprising about ten abundant nucleoid associated proteins (NAPS) with hundreds of target genes and more than a hundred small transcription factors with few targets (Marìnez-Antonio et al., 2008; de Matos Simoes et al., 2013). The actions of these regulators form the transcriptional regulatory network (TRN) of a bacterial cell. The TRN preferentially transmits information between different parts of the nucleoid, especially between macrodomains (Valens et al., 2004; Sobetzko et al., 2012) and thereby complements locally restricted regulation by DNA topology.

In summary, these regulatory mechanisms act alone or in combination on promoter activity and are, therefore, subsumed under promoter regulation in this manuscript.

In addition to this strategy, gene expression can be increased by adding more copies of a gene. This evolutionary strategy can be observed for highly transcribed genes like stable RNA operons, where promoter regulatory optimization is exhausted (Jinks-Robertson and Nomura, 1987; Condon et al., 1995) or fast adaptation to new environments beyond the intrinsic flexibility of an organism is required (Slack et al., 2006). Gene duplication does not alter individual promoter regulation unless titration of regulators to the increased number of binding sites is involved. As the majority of regulatory sites are covered by NAPS that bind hundreds of sites, a few additional sites usually have no relevant effect on the binding site to regulator ratio.

The increase in expression by adding copies of a gene takes place at an evolutionary time scale. However, there is also a mechanism for transient changes in copy numbers within the life cycle of a bacterial cell. During DNA replication, genes are either present in one copy in front of the replication apparatus or in two copies after replication of its locus. Hence, the closer a gene is to the origin of replication (oriC) the earlier it is copied. Consequently, it produces double the amount of RNA for a longer time period within the cell cycle than a gene located close to the terminus. Even with a maximum velocity of about 1000 bp/s for fast replicating bacteria (Huang and Ito, 1999; Egan et al., 2004), the time required for replication of the genome and preparation of cell division may extend beyond the doubling time (40 min compared to 20 min for *E. coli*) for fast growing bacteria. To overcome this limitation, fast growing bacteria turn to overlapping replication rounds, where new rounds of replication are initiated before the template DNA is fully replicated. This can increase gene copies up to 8 copies in *E. coli* in the oriC proximal region in comparison to the terminus region (Donachie, 1968; Bipatnath et al., 1998). Furthermore, this copy number effect is linked to the cell's doubling time. Under rich nutrient conditions, the copy number effect is maximal, whereas under conditions of starvation or stress no replication is initiated and locus copies are uniform along the chromosome (Ferullo et al., 2009). Earlier studies identified a link between gene expression of individual genes and their copy number (Chandler and Pritchard, 1975; Schmid and Roth, 1987; Sousa et al., 1997; Couturier and Rocha, 2006; Block et al., 2012; Bryant et al., 2014; Scholz et al., 2019). Two approaches were followed. The first approach focused on the investigation of a small fraction of native genes which gave a first indication that copy numbers may be involved in shifting expression levels for genes close to oriC. The second approach used the insertion of reporters to test the implications of insertion position on reporter activity. By using a single reporter the approach was not biased by the individual regulation of native genes. Therefore, the physical effect of copy numbers could be detected more clearly. However, this approach also had two major drawbacks: (1) Positional effects due to silenced regions, e.g. by H-NS may introduce local biases (Freddolino et al., 2021). Systematic errors could be introduced by potential DNA-supercoiling gradients along the chromosome due to a sensitivity of the reporter promoter (Lal et al., 2016; Klein et al., 2021). Furthermore, local transcription activity may add to reporter read-outs. Hence, results may be biased by specific sensitivities of the reporter system toward various signals. (2) The reaction of the reporter contains no information about the behavior of the native genes. Despite this pioneering work, no systematic analysis of the impact of copy numbers on the native gene expression system, functional regulation and its impact on chromosome plasticity has been performed yet. Hence, no conclusion about the impact on the regulatory system can be made. In 2013, we identified a gradient of activated genes following the oriC-ter axis (Sobetzko et al., 2013). This gradient covers the full chromosome and potentially influences the expression dynamics of many genes. In this study, we analyze and quantify the impact of copy numbers on forming a spatio-temporal expression pattern. We also quantify its impact on gene regulation of individual genes, functional groups and pathways. Furthermore, we show how copy numbers drive the arrangement of genes during evolution depending on species-specific growth rates.

## Results

### The spatio-temporal gene expression pattern between exponential and stationary phase might be explained by two different regulatory concepts

**Figure 1:**
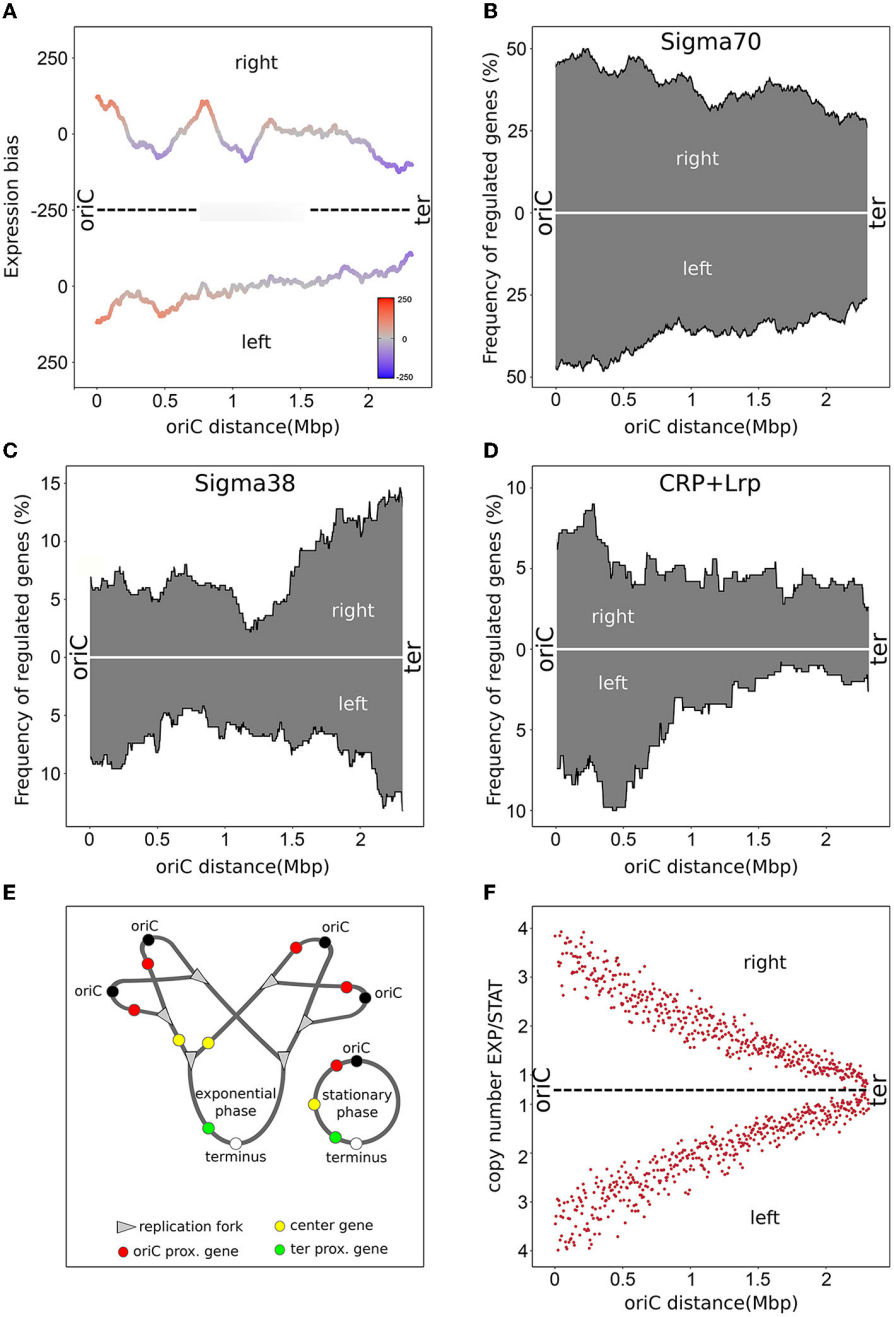
Overview of the *E*. coli wild type distributions and biases were calculated using a sliding window of 300 genes. Distributions were normalized over the total gene number of each window. The replichores (right/left) are organized from the left to the right representing the oriC and the terminus, respectively. **(A)** Spatial bias of up- and down-regulated genes between exponential and stationary phase in the *E. coli* CSH50 wild type. Transcriptomic data originated from Sobetzko et al. (2013) **(B)** Spatial frequency of Sigma70 regulated genes. **(C)** Spatial frequency of Sigma38 regulated genes. **(D)** Combined spatial frequency of CRP or LRP repressed genes. **(E)** Scheme of overlapping replication in exponential phase and its consequences on transient gene copies compared to stationary phase. **(F)** Marker frequency analysis of the *E. coli* MG1655 wild type strain for exponential phase normalized by stationary phase.

The comparison of the gene expression profiles in exponential and stationary phase reveals that the oriC-proximal genes have a higher expression in exponential than in stationary phase, while the terminus-proximal genes have a lower expression level (Sobetzko et al., 2013). A plot representation of the 2013 *E. coli* CSH50 wild type data is shown in Figure 1A. This spatio-temporal gene expression pattern may reflect a cellular program to adapt to changing conditions. The pattern may emerge due to the strategic positioning of genes regulated by global transcription factors such as σ^70^, σ^38^ or abundant regulatory proteins like Fis, H-NS, IHF, the cAMP receptor protein (CRP), or the leucine-responsive regulatory protein (Lrp). These factors regulate hundreds of genes and therefore may have an impact on a global pattern. A new analysis of the most recent regulonDB data set of target gene positioning of global regulators confirms a gradient of regulated genes in the σ^70^, σ^38^, CRP, and Lrp regulons along the oriC-ter axis (see Figures 1B–D and Supplementary Figure S6) found in Sobetzko et al. (2012). Activity of these factors depends on the cellular state. While σ^70^ is the dominant transcription factor during exponential phase, σ^38^ competes with σ^70^ for RNA polymerase (RNAP) during stationary phase (Jishage et al., 2002). The updated regulatory data of the regulonDB database confirmed genes specifically regulated by σ^70^ are more abundant in proximity to oriC (Sobetzko et al., 2012) (see Figure 1B), which may contribute to the observed increase of oriC-proximal genes during exponential phase. Genes regulated specifically by σ^38^, however, are more abundant at the terminus region (see Figure 1C), which can explain the observed increase of oriC-distal gene expression in stationary phase compared to exponential phase. CRP and Lrp, both important regulators during starvation and stationary phase (Schultz et al., 1988; Tani et al., 2002), regulate genes with a characteristic distribution gradient along the oriC-ter axis (see Figure 1D). In this case, repression of more oriC-proximal genes during stationary phase would also contribute to the observed increase in gene expression of oriC-proximal genes during exponential phase. In general, a combination of different regulatory proteins with non-uniform distribution of target genes along the oriC-ter axis contributes to the spatial gene expression pattern observed when comparing exponential and stationary phase. These patterns can be supported by DNA supercoiling sensitivity of promoters mediated by DNA structure and regulatory proteins (Blot et al., 2006; Sobetzko et al., 2012; Lal et al., 2016; Pineau et al., 2022).

Besides promoter regulation, which differs in its activity regarding growth phases, replication activity is another potential regulatory factor. During exponential growth, many bacteria perform multifork replication to ensure chromosome replication when the doubling time is shorter than the duration of replication (see Figure 1E). This resulting copy number effect leads to a higher expression of oriC-proximal genes during fast growth compared to slow growth or stationary phase. Toward the terminus region, this effect is gradually reduced. Marker-Frequency-Analysis (MFA) allows to visualize and quantify the copy number effect when using whole-genome DNA sequencing of exponential growing cells (see Figure 1F). For *E. coli*, a gradual decrease of reads along the oriC-ter axis is observed representing the average copy number of the sequenced culture (Skovgaard et al., 2011). However, both regulatory factors, promoter regulation and copy number effect, act in the same direction regarding increasing and decreasing oriC-proximal/distal genes during exponential and stationary phase. Therefore, it is only possible to determine the impact of each factor on gene expression by isolating a single factor.

### A strain to dissect the impact of promoter regulation and copy number effects

To determine the influence of promoter regulation and the copy number effect on the gene expression pattern of exponential phase compared to stationary phase, one factor has to be altered and the other has to be kept constant. As changes in the genetic regulatory network would be difficult due to the diversity of regulatory proteins and DNA topology (Sobetzko, 2016; Martis B et al., 2019; Klein et al., 2021), we decided to significantly alter the copy number effect. By relocation of the oriC into the terminus region, one would expect an opposite copy number profile compared to wild type. In exponential phase, genes initially located in the ter domain in wild type would have a higher copy number than genes initially located in the oriC-domain in wild type. This inversion of gene copy number could result in an unaltered, a disturbed or an inverted expression profile when compared to stationary phase depending on the impact of each of the regulatory factors. However, relocating the oriC into the terminus can cause massive biological problems. In the terminus region, the replication forks are trapped at specific DNA sites called ter sites, which are bound by the terminus utilization substance protein (Tus) (Hill, 1992). This protein-DNA complex unidirectionally arrests DNA replication, allowing replication forks to pass ter sites only in the origin-to-terminus direction. An oriC in the terminus region would, therefore, lead to replication fork stalling shortly after initiation and prevent replication of the remaining chromosome. To circumvent this problem, we generated an *E. coli* MG1655 Δtus strain to abolish replication stalling at ter sites. This allowed the replication forks to pass freely from the former wild type terminus to the former oriC region.

Another problem is head-on replication-transcription conflicts of the highly transcribed ribosomal RNA (rrn) operons and the replication forks, as the *rrn* operons are transcribed in oriC-ter direction. These head-on collisions seem to significantly delay fork progression and especially the *rrnCABE* cluster and the *rrnH* operon causes substantial problems to replication progression (Ivanova et al., 2015). Therefore, we needed to alter the transcription direction of the *rrn* operons. The Cre-lox and FLP/FRT systems, which are based on site-specific recombinases, allow excision and inversions of chromosomal DNA flanked by two identical target sites depending on its relative orientation. By flanking *rrn* operons with facing FRT or loxP site pairs, it is possible to invert the transcription direction and circumvent head-on replication-transcription machinery collisions, when relocating the oriC to the terminus region. To minimize crosstalk between FRT/loxP sites of different inversion sites, different FRT/loxP variants were used for each pair (Missirlis et al., 2006; Turan et al., 2010). As the *rrnCABE* cluster consists of four nearby ribosomal operons in oriC-proximity, we only used one pair of FRT sites to invert the whole region instead of inverting every single operon on its own. All insertions and deletions were carried out using the CRISPR SWAPnDROP system, which allows consecutive chromosomal changes based on CRISPR/Cas9 counter-selection (Teufel et al., 2022).

For the relocation of the oriC into the terminus, we first replaced the native oriC with the F-plasmid origin of replication oriS flanked by a pair of tandem FRT sites to allow excision of the oriS at a later stage using the FLP/FRT system. This also allows for a parallel inversion of the ribosomal RNA operons together with the oriS deletion in a single step to avoid head-on collisions in intermediate strains in case of a sequential genome editing approach. After the replacement of oriC with oriS, we inserted the native oriC into the terminus region of the chromosome. The strain was then transformed with a plasmid harboring the Cre recombinase and Flippase under the control of the pBAD promoter. Additionally, we used CRISPR/Cas9 to actively remove oriS DNA looped-out during excision by FLP/FRT-recombination to prevent reintegration of generated mini replicon. In summary, the final strain was able to freely invert its *rrn* operons and remove the placeholder oriS upon induction of the CRISPR/Cas9 and recombination systems to generate a strain with an inverted copy number gradient.

**Figure 2:**
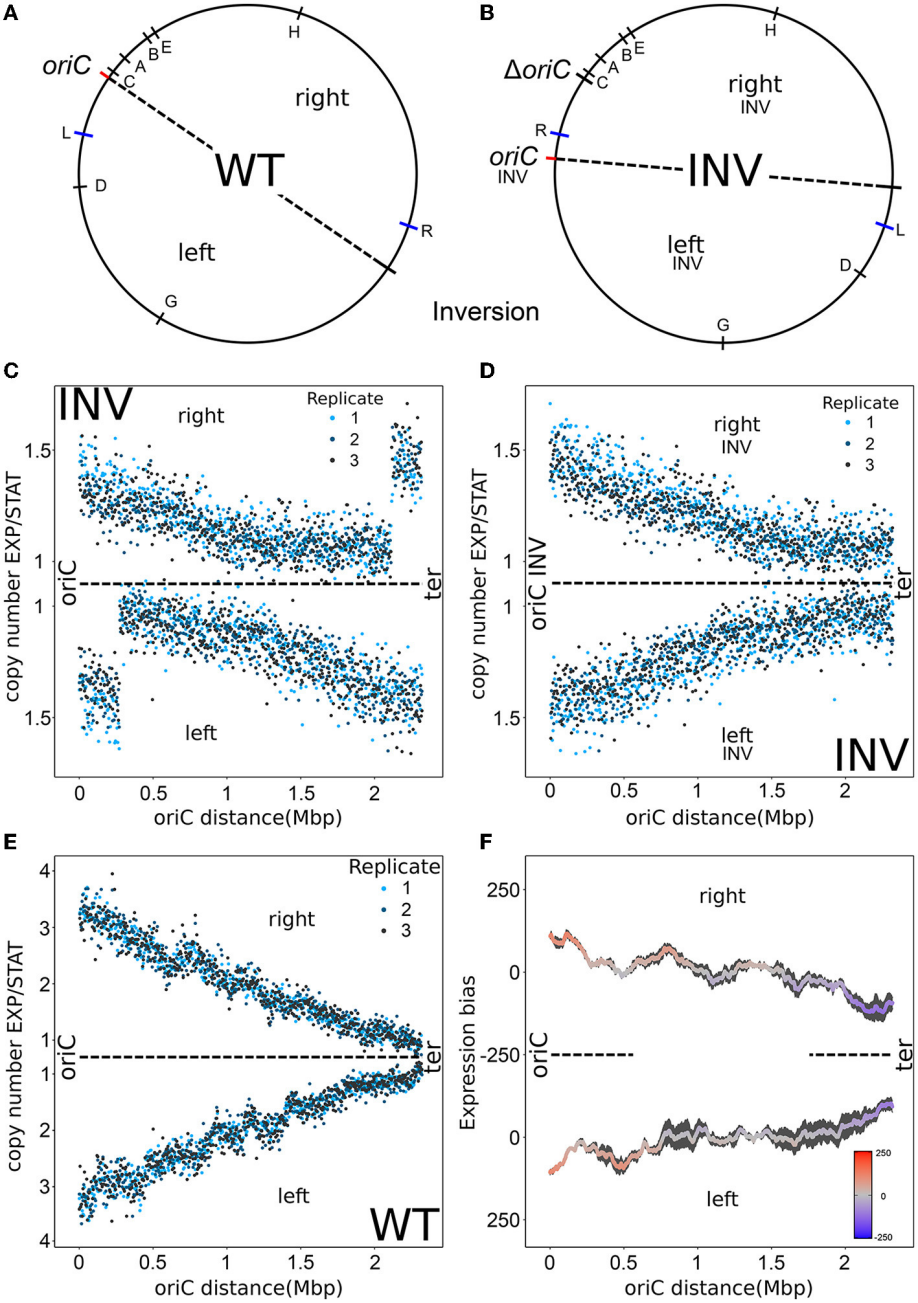
Characterization of the INV and WT strain. **(A)** Chromosomal map of the WT strain. The inversion region for the derived INV strain is indicated in a red to blue gradient. Ribosomal RNA operon positions are indicated by capital letters within the circle. The letters L and R outside the circle indicate inversion break points. The dashed line indicates the chromosomal symmetry axis. **(B)** Chromosomal map of the INV strain with the same indications as in **(A)**. **(C)** Marker frequency analysis of the INV strain for exponential phase normalized by stationary phase mapped against the WT genome. **(D)** Marker frequency analysis of the INV strain for exponential phase normalized by stationary phase mapped against the INV genome. OriC INV, right_*INV*_, left_*INV*_ indicate the new chromosomal organization of the INV strain. **(E)** Marker frequency analysis of the WT strain for exponential phase normalized by stationary phase. **(F)** Spatial bias of up- and down-regulated genes of three replicates between exponential and stationary phase in the WT strain using a sliding window of 300 genes. Standard error is indicated by a black area around the mean.

After induction, streaked colonies appeared in different sizes ranging from very small to wild type-like size. OriS elimination could only be found in small and middle-size colonies. Surprisingly, *rrn* operon inversions occurred rarely and could not be found in combination with the oriS elimination. Colonies of different sizes and with oriS knockout were re-streaked for further investigation. Re-streak of the small-size colonies resulted in a mix of small and middle-sized colonies indicating instability of the strain due to frequent suppressor mutation. For stability reasons, we used one of the middle-sized colonies that originated from a re-streaked small-size colony for further investigations. These colonies remained stable and homogeneous with respect to colony size and morphology for several restreaks. MFA-analysis of the clone during exponential phase revealed an inversion spanning half of the chromosome, mainly the left replichore (see Figures 2A–D). This inversion resulted in a relocation of the oriC from the terminus back into the wild type oriC region with a distance of about 381 kb from the native oriC site. Thereby, for most right *Replichore* genes, a wild type oriC distance configuration was restored whereas most genes of the left replichore remained inverted with respect to oriC distance. Furthermore, for the *rrnCABE* cluster and the *rrnH* operon the wild type leading strand arrangement was restored and therefore head-on collision with replication was prevented. This might have improved fitness and explains the increased colony size (Ivanova et al., 2015). Additionally, this strain revealed a significantly reduced maximal copy number at oriC during exponential phase compared to wild type (see Figures 1F, 2D). Consistently, the doubling time of this inversion strain (INV) at around 62 minutes is three times greater compared to the wild type (see Supplementary Figure S1A).

With its stability and the strongly reduced copy-number in exponential phase, this strain can be used for further investigation of copy number impact on the spatio-temporal expression pattern. If copy numbers have a strong impact, comparison of the exponential and stationary phase should show a reduced or abolished expression gradient along the oriC-ter axis. For the reference strain, we decided not to take the wild type *E. coli* MG1655 parental strain but a strain containing all the modifications leading to the INV strain to avoid potential effects of the modifications when comparing to the INV strain. The strain prior to induction of recombinases and CRISPR/Cas9 was selected. It shared all modifications except for the inversion and the presence of oriC in the terminus region as well as the oriS in the native oriC location. In order to obtain the reference strain, the terminus oriC was removed and the place-holder oriS at the native oriC site was replaced by oriC to recover the wild-type origin of replication. This reference strain finally resembled the wild type genome arrangement and at the same time shared the modifications required to generate the INV strain (see Supplementary Figures S1A, B). The fitness and integrity of the reference strain was then tested and compared to the *E. coli* MG1655 parental strain. Concerning fitness and growth speed (19.6 min doubling time) , the reference strain and *E. coli* MG1655 showed no differences. MFA-analysis showed a replication profile similar to *E. coli* MG1655 regarding spatial copy number distribution (see Figures 1F, 2E). Comparison of RNA-seq data of *E. coli* MG1655 and the reference strain showed a similarity in gene expression that equals the similarity of replicates of a single strain (see Supplementary Figures S7C, D). Hence, from the perspective of mRNA levels, the reference strain cannot be distinguished from its wild type precursor *E. coli* MG1655. In analogy to the RNA-seq data analysis of *E. coli* CSH50 wild type (Sobetzko et al., 2013), a sliding window approach was used to determine spatial biases of up/down regulation (see Section Material and methods). RNA-seq analysis of the exponential and stationary phase revealed the same spatio-temporal gene expression pattern seen in the reference study (see Figures 1A, 2A). Due to the high degree of similarity of the reference strain and the *E. coli* MG1655 parental strain, the reference strain is referred to as wild type (WT) for the rest of the manuscript.

### Copy number is the dominant effect of the spatio-temporal expression pattern

**Figure 3:**
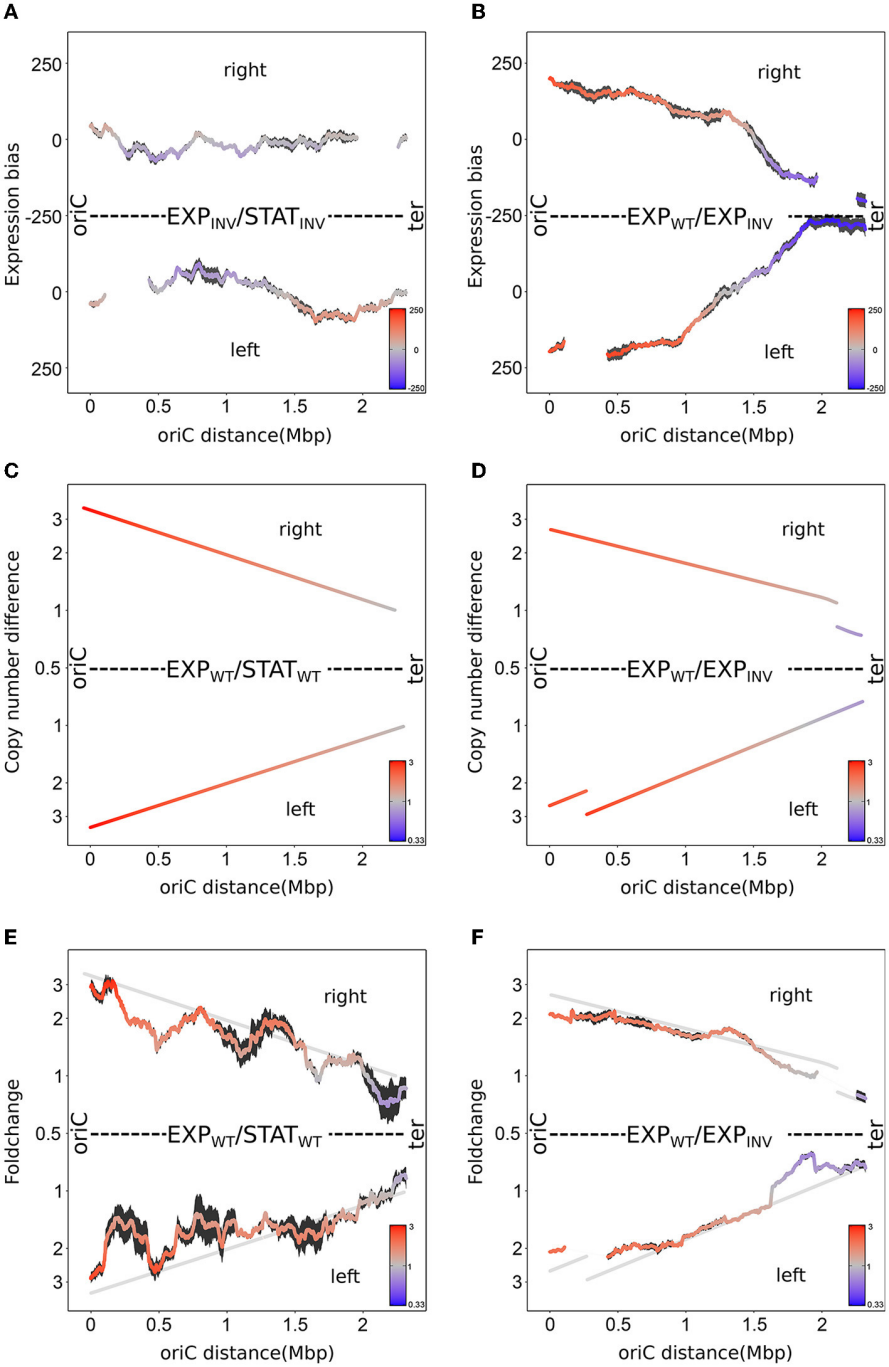
Impact of copy number on chromosomal spatial gradients. **(A)** Spatial bias of up- and down-regulated genes (sliding window of 300 genes) of three replicates between exponential and stationary phase in the INV strain mapped against the WT genome. Standard error is indicated by a black area around the mean. **(B)** Same as in **(A)**, but for the comparison of WT and INV strain during exponential phase. **(C)** Average local difference in copy number derived from MFA analysis of three replicates between exponential and stationary phase in the WT strain. **(D)** Same as in **(C)**, but for the comparison of WT and INV strain during exponential phase. **(E)** Average local expression fold change (sliding window of 300 genes) of three replicates between exponential and stationary phase in the WT strain. The data was normalized for relative frequency biases (see Section Material and methods). The gray line indicates the local copy number differences of **(C)**. **(F)** Same as in **(E)**, but for the comparison of WT and INV strain during exponential phase. The gray line indicates the local copy number differences of **(D)**.

For comparison of WT and INV transcription patterns, RNA-seq of the INV and WT strains was carried out in triplicates. In analogy to WT, we analyzed the expression profile of the INV strain in the exponential (EXP) and stationary (STAT) phase. For better comparison, the expression profile was mapped against the WT chromosome. Mapping against the INV chromosome would alter the coordinate system of the chromosome with respect to WT comprising altered replichores and inverted regions. Comparison and interpretation of the data would therefore be difficult. As a consequence of the sliding window approach, windows spanning the inversion break points are not present in both data sets and are therefore excluded from the analysis (see Section Material and methods and Figure 3A). As seen in Figure 3A, the spatial expression gradient of the INV strain along the oriC-ter axis is mostly attenuated compared to WT. Nevertheless, local characteristic peaks of the spatial pattern are still consistent with the wild type pattern suggesting a state of the promoter regulatory system similar to WT (see Supplementary Figure S2). This indicates that copy number effects may play a major role in the formation of the gradual expression pattern while promoter-specific regulation remains unchanged. More compellingly, for the left replichore in Supplementary Figure S3A, genes closer to the terminus show a higher expression in exponential phase compared to the stationary phase. Due to the inversion, these genes are situated close to oriC_*INV*_ in the INV strain. Although general copy number is reduced significantly due to the slower growth rate, the effect of inverted copy numbers in the inversion region is still reflected in the expression profile in the INV strain.

To verify the effects of copy number and study them isolated from other regulatory effects, exponential phase of WT and INV strains were compared in the same growth phase. While both strains differ in strength and orientation of the copy number, in the same growth phase, differences in promoter regulation are expected to be minimal. The comparison revealed a very strong expression bias gradient along the oriC-ter axis (see Figure 3B). The vast majority of oriC-proximal genes show a higher expression in the WT strain, while genes closer to the terminus are predominantly higher expressed in the INV strain. Interestingly, in the putative absence of difference in promoter regulation, the gradient is even more pronounced than between the exponential and stationary phase indicating that the copy number effect is the source of the gradual spatial expression pattern. Additionally, the characteristic local peaks seen in the analysis of WT EXP/STAT and INV EXP/STAT cannot be observed. This underpins the promoter regulatory origin of the local peaks between the exponential and stationary phase. Furthermore, the expression biases of both WT EXP/STAT (see Figure 2F) as well as the WT/INV EXP (see Figure 3B) follow their corresponding copy number differences (see Figures 3C, D). The expression bias even reflects the small steps in copy number differences at the inversion break points (see Figures 3B, D). Due to the inversion of the major part of the left replichore in the INV strain, copy number differences between WT and INV strain are not symmetrical on both replichores. The impact of the inverted copy number on the left replichore of the INV strain is rather small due to the relatively flat overall copy number gradient of the INV strain (see Figure 2C). However, the impact of the reverted copy number of loci on the left replichore is causing in a steeper copy number difference gradient of the left replichore compared to the right replichore in the comparison of WT/INV during exponential phase (see Figure 3D). The steeper gradient is also reflected in the gene expression bias gradient (see Figure 2F), indicating a close relation of gene copy numbers and the gene expression bias.

Even though the previous data suggest a major role of the copy number effect on the expression profile, the exact impact is not yet quantified. Whether other regulatory factors systematically contribute positively or negatively to the spatio-temporal expression pattern is still an open question. Multiple copies of a gene cause an increase in gene expression proportional to the number of copies. Consequently, the average expression fold change should match the corresponding copy number differences, if the copy number effect is the dominant factor. If other systemic regulatory factors influence the spatio-temporal pattern, the average expression fold change should deviate significantly from the copy number difference. As seen in Figure 3E, the average expression fold change of WT EXP/STAT corresponds well to the copy number difference (see Figure 3C). For the case of WT/INV EXP (see Figures 3D, F), where copy numbers were systematically reduced in the INV strain, the fold changes also matched the copy number differences supporting the role of copy numbers in forming spatial expression patterns. In this case, the characteristic local peaks (see Supplementary Figure S2) observed between exponential and stationary phase is flattened out, indicating a promoter regulatory source between these phases.

In certain cases, it is important to remove copy number effects from expression data. Such cases are mutant studies in which regulatory effects of the mutant are investigated. A growth defect often observed in regulator mutants would introduce a bias caused by copy number differences between wild type and mutant (see Supplementary Figure S5) (Beber et al., 2016). Consequently, gene expression data and deduced regulatory interactions might be biased. Therefore, we tested this scenario by first normalizing the WT exponential phase expression data by the copy number difference between WT and INV in exponential phase. If copy numbers are a major player, the removal of the copy number effect in WT with respect to INV should generate an expression pattern similar to INV in exponential phase. Consequently, EXP_*WTcor*_/STAT_*WT*_ should resemble EXP_*INV*_/STAT_*INV*_. The comparison revealed a remarkable similarity (see Supplementary Figure S4). This shows that copy number data can be used to compensate for copy number differences between samples and underpins the impact of copy numbers on forming spatial expression patterns.

### Copy numbers regulate distinct cellular functions

We have shown that the copy number effect plays a major role in forming a spatio-temporal gene expression pattern between exponential and stationary phase. This may also indicate a central role in the regulation of individual genes and pathways. However, a spatial bias induced by copy numbers does not necessarily imply a major role in single gene regulation. Promoter regulation may alter gene expression several hundred-fold (Lu, 1997). Regarding total fold change, the fold change of copy number can be neglected in such cases. To estimate the impact of copy number relative to other regulatory factors, we analyzed the single gene expression fold change data of WT EXP/STAT. The expression fold change of a gene is determined by its difference in copy number and promoter regulation. We have shown that on a large scale (300 genes average), the expression fold change follows the copy number between exponential and stationary phase. Therefore, we can remove the copy number effect (*f*_*copy*_) of single genes from its expression fold change (*f*_*total*_) to determine the influence of the remaining promoter regulation (*f*_*reg*_).


(1) $$\begin{array}{l} f_{reg}=\frac{f_{total}}{f_{copy}} \end{array}$$

**Figure 4:**
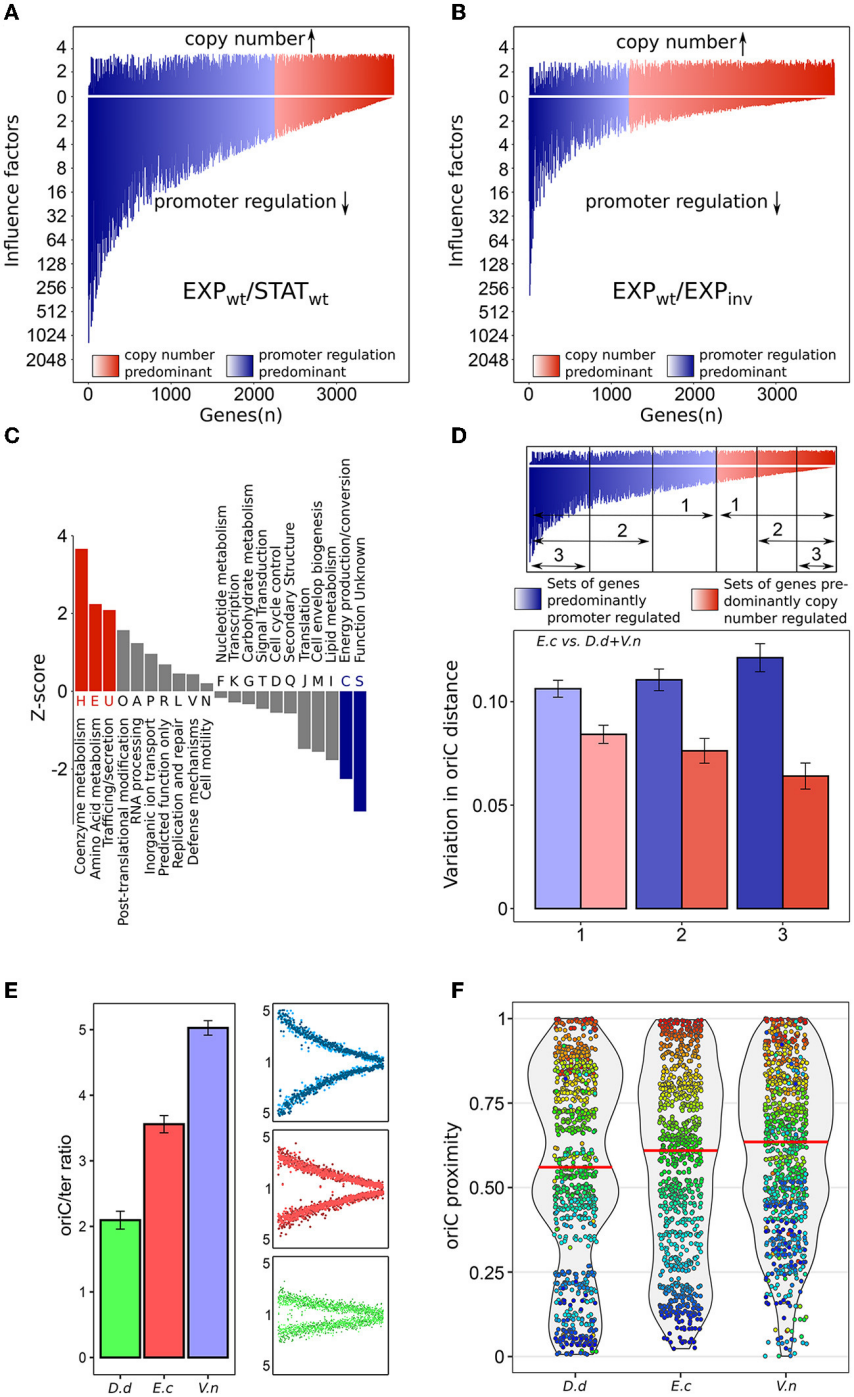
Impact of copy number on gene regulation and evolutionary gene migration. **(A)** Copy number factor and promoter regulation factor of all genes sorted by its ratio in the comparison of wild type between exponential and stationary phase. Rightmost genes show the highest impact of copy number effect on its total regulation. Blue colors indicate a higher impact of promoter regulation whereas red colors indicate a higher impact of copy number regulation. **(B)** Same as in **(A)**, but for the comparison of WT and INV strain during exponential phase. **(C)** Significance (z-score) of over- and under represented functional groups of WT genes predominantly regulated by copy number for the comparison of exponential and stationary phase. **(D)** Conservation of oriC distance of *E. coli* orthologous gene present in *D. dadantii* and *V. natriegens*. Variation is the fraction of the full oriC-ter distance. Red and blue colors indicate the sets of predominantly copy number and promoter regulated gene sets with different stringency, respectively. **(E)** Copy number of *D. dadantii, E. coli* and *V. natriegens* and the corresponding marker frequency plots for exponential growth. **(F)** oriC distance violin plots with orthologs of *D. dadantii, E. coli* and *V. natriegens*. Orthologous genes are active during exponential growth in *E. coli*. Median values are indicated by horizontal red lines. Individual genes and its orthologs are indicated as dots and are color coded according to the oriC-ter order in *E. coli*.

We compared the copy number influence factor and the promoter influence factor of each individual gene and divided them into two categories: predominantly copy number regulated and predominantly promoter regulated, depending on the proportion of each factor on the total fold change of gene expression. About 40% of the genes are predominantly regulated by copy number when comparing exponential with stationary phase (see Figure 4A). For more than three quarter (78%) of genes, its copy number still covers more than one fourth of its total fold change. This underlines the broad relevance of the copy number effect in gene expression. However, there are also genes which have a significantly higher fraction of promoter regulation. For those genes, the copy number effect presumably plays a minor role in regulation.

When comparing the influence factors of WT/INV EXP, the majority of the genes (67%) are predominantly regulated by the copy number effect with a reduced influence of other regulators (see Figure 4B). In this case, about 92% of the genes have a copy number influence factor, which is greater or equal to one fourth of the influence factor of other regulators. This reflects the mild influence of promoter regulation and the copy number dominance in this experimental design. The remaining fraction of altered promoter regulation could originate from the altered expression of regulators including RpoS, Fis, Crp, or StpA located in the inversion region of the INV strain and its secondary effects.

The large set of genes dominantly regulated by copy numbers may indicate a concerted regulatory mechanism. In the case of WT/INV EXP, the experimental setup was rather artificial and did not follow a process in the life cycle of *E. coli*. Therefore, we focused on the WT EXP/STAT experiment to see if there is a link between regulation by copy number and specific cell functions. For genes predominantly regulated by the copy number, frequencies of functional categories were investigated (see Figure 4C). We found a significant over representation of genes in the “Coenzyme metabolism”, “Amino acid metabolism”, and “Trafficking/secretion” categories, while in “Energy production and conversion” genes regulated by copy number were under represented. For coenzyme metabolism, it was shown that in *E. coli* coenzyme synthesis is directly correlated to growth (Hartl et al., 2017). *E. coli* is capable to effectively adjusting de novo coenzyme synthesis to counteract varying dilution rates during growth, but the regulation is still unknown. Hence, a direct linking to replication rounds and therefore to copy number appears plausible. Also, amino acid metabolism is involved in biomass formation and is, therefore, a plausible candidate for coupling to copy numbers. Analysis of individual metabolic pathways further supports a coherent regulation by copy numbers. Here, specific pathways were strongly enriched in copy number dominated genes e.g., the aspartate pathway (see Supplementary Figure S3). Interestingly, in this pathway, the copy number regulation of intermediate pathway steps is complemented by promoter regulation at key steps at the entry and exit points. Lists with all metabolic pathways from *E. coli* and the corresponding copy number influence, as well as raw data for fold change analysis, can be found in the Supplementary material.

### Regulation *via* transient copy numbers determines chromosomal architecture in the course of evolution

As coenzyme and amino acid metabolism are essential in bacteria, their genes are evolutionary conserved. With respect to their regulation by copy number, those genes may also exhibit conservation regarding the location on the chromosome. Genes, which are coupled to growth and copy number should be located close to the oriC or at least maintain their relative position to oriC. In order to investigate the evolutionary conservation of genes predominantly regulated by copy number, we first divided all genes into different sets depending on the extent of copy number regulation (see Figure 4D). Three opposing sets were generated with increased stringency for either copy number or promoter regulation dominance. We then estimated the variation of those genes in two species (*Dickeya dadantii* and *Vibrio natriegens*) with respect to their oriC distance in *E. coli*. *D. dadantii* is part of the Enterobacterales and is the causative agent of bacterial stem and root rot affecting potatoes and other crops, while *V. natriegens* is part of the Vibrionales and of increasing biotechnological relevance. The stronger the gene regulation is dominated by the copy number, the less variation in oriC distance is observed in these two species compared to *E. coli*. In contrast, the stronger their regulation is dominated by promoter regulation, the more variation is detected. This indicates an oriC distance conservation of genes regulated by copy number and high spatial flexibility of genes governed by promoter regulation. This result refines the general observation of conserved positioning of genes with respect to the oriC distance in bacteria (Sobetzko et al., 2012).

**Figure 5:**
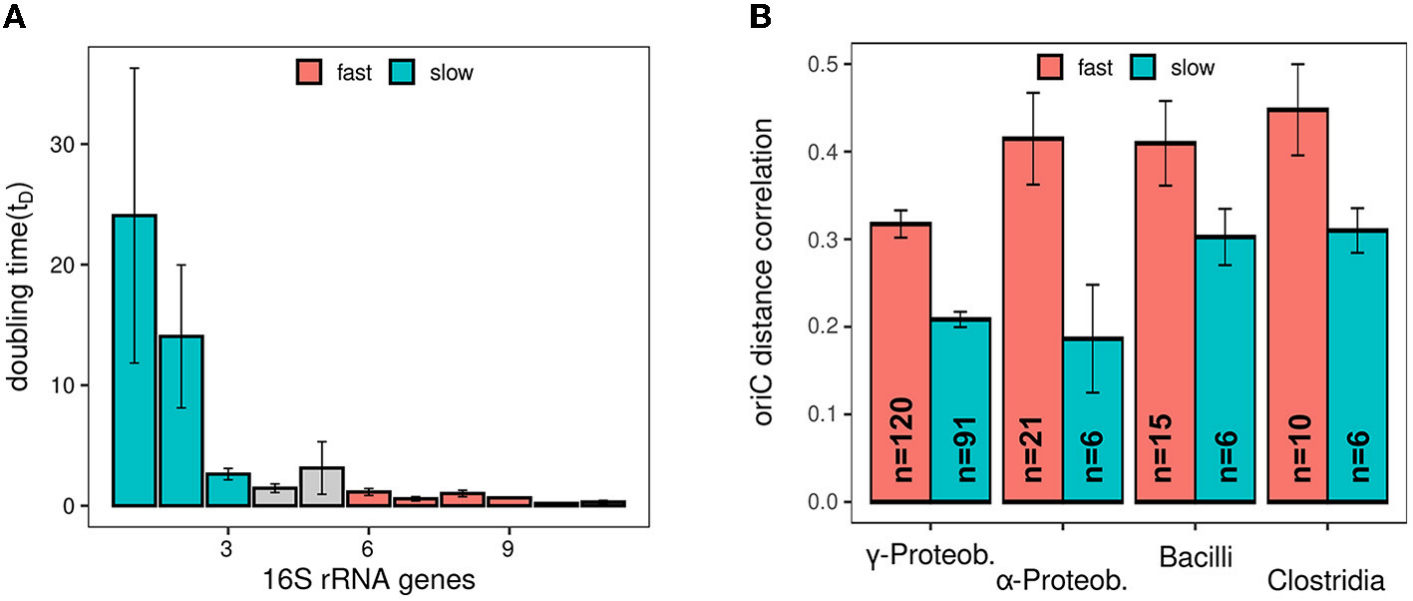
Analysis of oriC distance conservation in slow and fast growing bacteria. **(A)** Interdependence of doubling time and number of 16S rRNA genes. Selected groups for fast and slow growing species are indicted by red and blue. **(B)** Average oriC distance correlation of species from different classes. The number of pairs used for averaging are indicated in the bars. Red and blue colors indicate the groups of fast and slow growing species.

Selective pressure for copy number regulation could depend on the extent of the available copy number effect. Consequently, for faster growing species, a higher portion of genes active during exponential growth could exploit copy number effects for regulatory purposes and migrate toward oriC. The two selected species flank *E. coli* with respect to doubling time during exponential growth. *D. dadantii* (~100 min) has a longer doubling time than *E.coli* (approx. 20 min) whereas *V. natriegens* (approx. 10 min) exhibits a far shorter doubling time. As DNA polymerase speed is a limiting factor for fast growing bacteria, a reduced doubling time is reflected in intensified overlapping replication increasing copy numbers (see Figure 4E). Consequently, in these species, three different levels of copy numbers are realized allowing us to investigate the impact of copy numbers on chromosome evolution with respect to gene location. For comparison of the three species, genes predominantly expressed during exponential phase in *E. coli* (p-value <0.05) were selected. Due to the difference in expression between exponential and stationary phase, copy number can potentially positively regulate these genes also in other organisms. The distribution of orthologs in the three species reveals a link between growth rate and stringency of gene positioning (see Figure 4F). Orthologs in the slow growing bacterium *D. dadantii* were less focused on the oriC proximal region than orthologs in *E. coli*. Orthologs in *V. natriegens*, the fastest growing bacterium, were even more focused in the oriC proximal region than in *E. coli*. We further investigated this observation using a larger set of species. For most species, the oriC position is not determined (Gao and Zhang, 2007) or hidden in countless publications. Therefore, we devised a method that is based on the oriC-ter symmetry found across the bacterial kingdom (Sobetzko et al., 2012). Hence, the chromosomes of two species match best with respect to oriC distance if oriCs of both species are superimposed (see Supplementary Figure 8 and Section Material and methods “constellation analysis”). Using this approach, oriC distance correlation was determined for species pairs of various phylogenetic classes including gram-positive and gram-negative bacteria. The species within these classes were selected to be different in their family membership to ensure a standardized evolutionary distance. To approximate growth rates, we used the correlation of growth rate and the number of ribosomal operons of a species. This correlation was first verified using growth rates of Couturier and Rocha (2006) and NCBI 16S rRNA annotations (see Figure 5A). Species in each class were split into slow growing (16S rRNA ≤ 3) and fast growing (16S rRNA ≥ 6). For all species pairs in these sets, oriC distance correlation was determined. Consistent with the initial analysis in Figure 4F, fast growing species showed a stronger correlation of oriC distance between its orthologs than slow growing species in all investigated classes (see Figure 5B). This indicates that copy number regulation is also involved in the evolutionary shaping of bacterial chromosomes proportional to its regulatory potential.

## Discussion

In this study, we obtain new insights into spatio-temporal regulation in bacteria caused by replication-induced chromosomal copy number effects. We could show that the gene expression pattern observed when comparing exponential and stationary phase can be explained by copy number differences between the two growth phases instead of spatio-temporal promoter regulation.

The initial approach was the construction of a strain, which harbors inverse copy numbers due to the relocation of the oriC into the terminus. However, moving the oriC causes conflicts with several cellular systems coupled to replication. Conflicts include the directional tus/ter replication termination system that block replication from the terminus toward the native oriC location. Therefore, we tried to induce rRNA operon inversions by flanking loxP and FRT sites. Other non-essential systems were not altered to reduce further invasive chromosome modifications. Systems like nucleoid occlusion (SlmA) (el-Hajj et al., 1988), oriC macrodomain formation (maoP/maoS) (Valens et al., 2016) and ter domain formation (matS/matP) (Mercier et al., 2008) depend on strategic positioning of binding sites. Transplantation of the sites to the new oriC or ter sites would have caused massive genome modifications potentially disrupting the local sequence context with unpredictable effects on chromosome integrity or transcription proximal to the deletion and insertion sites. Although not essential (Blakely et al., 1991), we attempted to transfer the dif site to the native oriC locus but were not able to obtain positive clones. However, the resulting strain was viable, but showed frequent mutants with higher fitness. Genome analysis of a mutant revealed a large inversion covering the left replichore. The inversion recovered replication direction of the right replichore with respect to wild type. This suggests a strong preference to maintain the orientation of the rRNA operons mainly located on the right replichore. Studies with altered oriC positions showed similar rearrangements to avoid head-on collisions (Ivanova et al., 2015). The inversion also partially restored high copy numbers of these operons found in wild type by moving oriC in close proximity and at the same time abolished head-to-head collisions of RNAP and DNAP. A connection to macrodomains or overall chromosome structure is unlikely as the inversion disrupts the ori and ter macrodomains. Other chromosomal organization systems such as nucleoid occlusion (SlmA) or terminus segregation FtsK/KOPS usually symmetric to the oriC-ter axis are also disrupted by the inversion of a single replichore. This might be an indicator that these systems would have shown a minor contribution to improving the design of the initial approach with fully inverted copy numbers. The question remains, why no inversion of a rRNA operon was observed. Initial experiments to test the Cre and FLP systems showed such inversions in the rRNA operons. We can therefore assume that the systems were functional. Furthermore, the removal of the oriS in the native oriC locus showed that the Cre and FLP system were functional. It is likely that the distortion of the transcriptional regulatory system by altered copy numbers may be an important factor in the selection for inversion as the inversion at least recovered copy numbers for the right replichore. On the right replichore many major regulators controlling stationary phase like Dps LRP and H-NS are situated. These regulators were close to the relocated oriC in the initially intended fully inverted strain before the inversion found in INV. In this case, stationary and exponential regulation may collide slowing down growth (McGovern et al., 1994) and selecting for an inversion. However, the strain harboring the inversion (INV) still shows an increased doubling time (61.5 min) compared to WT (19.6 min). This can be due to a combination of the altered transcription due to gene copy changes and the head-on collisions of RNAP and DNAP due to the remaining inversion of the left replichore relative to wild type. Nevertheless, the strain was stable despite showing a reduced copy number gradient compared to WT and met the requirements of the study. In particular, the difference in copy number compared to WT allowed us to isolate the copy number effect from other regulatory factors and investigate the global expression pattern. Expression analysis showed that, except for expected differences due to modified copy numbers, the overall mRNA levels were mainly consistent with WT expression in exponential phase (see Supplementary Figures S2, S4). This indicates a regulatory state without global perturbances.

When comparing the exponential and stationary phase of the INV strain, the oriC-ter gradient in the expression pattern is more flat than in WT giving the first indication of the major role of the copy number effect as a global regulator. By comparing the exponential phases of WT and INV, we were able to eliminate the effect of promoter specific regulation as the same growth phase was compared. The analysis reveals a strong gradient in the expression pattern resulting from the differences in copy number between the strains. This is consistent with the results of reporter genes placed around the chromosome (Sousa et al., 1997). Furthermore, computational normalization of the wild type expression with respect to individual gene copy numbers generated a pattern resembling the INV strain expression pattern, where gene copy numbers were strongly reduced by the reduced growth rate (see Supplementary Figure S4). Quantification of the average fold changes between exponential and stationary phase in WT tightly followed the measured copy numbers. This proved the general ability of the copy number effect to produce the distinctive expression pattern we observed in the WT strain. Given the good correlation, other influence factors based on promoter regulation including sigma factors, NAPS and DNA supercoiling gradients suggested in other studies to play a role in forming a spatio-temporal gradient (Sobetzko et al., 2013) seem to play a minor role in forming the expression gradient from oriC to ter. Nevertheless, sigma factors and NAP binding as well as DNA supercoiling gradients follow a symmetric pattern on both replichores (Sobetzko et al., 2013; Lal et al., 2016; Visser et al., 2022). This is likely due to the selection on gene migration to favor increased gene copy number effects observed in this study. According to our current study, promoter regulation mainly influences the spatial pattern on a more local scale. On top of the general copy number pattern, characteristic local peaks were present in the wild type and INV strain highlighting the importance of specific promoter regulation. This conclusion supported by the comparison of WT and INV both in exponential phase in which the promoter regulation differences are expected to be minimal. Consequently, the characteristic local peaks were not present. This is consistent with Scholz et al. (2019) who reported characteristic regions with high or low expression during exponential phase using high-throughput reporter insertions. These regions were associated with NAP binding preferences. The analysis of major NAP binding site distribution further support this view. This rather dispersed action of classical regulators throughout the chromosome is consistent with the non-local nature of the regulatory network (Kosmidis et al., 2020). Interestingly, Scholz et al. (2019) observed a gradient of DNA accessibility by RNAP along the oriC-ter axis even after normalizing for the copy number bias in exponential phase. As copy numbers very well match the expression changes between exponential and stationary phase in our study, this accessibility bias could be a constant bias throughout growth phases and is therefore not visible in a comparison of two growth phases. A closer analysis may reveal a underlying conserved 3D DNA structure or hidden properties of DNA for RNAP accessibility.

Although fold change sliding window averages strictly follow the copy number, single genes can still strongly deviate positively or negatively from the average, but cancel each other out during averaging. Therefore, promoter regulation may not play a crucial role in global spatial pattern formation but can still dominate the regulation of single genes. To investigate regulation on the single gene level, we decomposed the fold change of each gene into a copy number and a promoter regulation component. We tested this analysis by comparing the exponential phase of the WT and INV strain, which revealed that most genes are predominantly copy number regulated as expected when promoter regulation differences are minimal (see Figure 4B). The remaining promoter regulation derived from differences between the two strains e.g. the inversion of the left replichore and its secondary effects. For the WT growth phase transition from exponential to stationary phase, about 40% of the genes still showed a dominant copy number regulation and even 75% of the genes were at least controlled to 25% by copy numbers. These numbers indicate, that the copy number effect acts as a regulatory principle that can be compared with other major regulators like the transcriptional regulatory network (Marr et al., 2008), major sigma factors and DNA supercoiling (Blot et al., 2006). These can control specific cellular functions and thereby contribute to a coherent organization of the cell. To test for a putative specific regulation of the copy number effect, predominantly copy number regulated genes were investigated with respect to their abundance in various functional groups. In agreement with (Couturier and Rocha, 2006), we found genes involved in transcription and translation enriched in the copy number regulated genes. A detailed analysis of all functional groups revealed further groups associated with copy numbers. A significantly high frequency of genes was involved in coenzyme metabolism and amino acid metabolism known to be related to growth (Hartl et al., 2017; Zampieri et al., 2019). Processes like these, directly coupled to cell growth and division, by their nature, require fold changes in gene expression in the range of growth rates that can be reached by *E. coli*. Transient copy numbers are linked to the growth rate by DNA replication frequency. Consequently, such processes are prone to be regulated by transient copy numbers. On the other hand, genes in the group of energy metabolism were under represented. Although this sounds counter-intuitive, energy metabolism depends on the presence of various distinct molecular sources of energy (Stülke and Hillen, 1999; Zampieri et al., 2019). Consistently, promoter regulation based on sensing these specific compounds and in turn activate specific pathways, is more pronounced for this set of genes and copy numbers play a minor role. Hence, processes requiring complex regulation or higher fold changes are predominantly regulated by other regulatory mechanisms like transcription factors. Such a case was reported by Chandler and Pritchard (Chandler and Pritchard, 1975) with respect to the tryptophane synthesis. Here the synthase was independent of the DNA content within the cell, Hence, DNA copy numbers were not the driving force of tryptophane synthesis. Other feedback mechanisms related to cell mass and availability of tryptophane were involved in controlling the pathway output. Interestingly, a combination of both regulatory concepts can be observed in the aspartate pathway (see Figure 3). Here, the basal level of the pathway is controlled by copy numbers and the internal balance of alternate fluxes is modulated by promoter regulation. Another indicator of coherent regulation by copy numbers is the conservation of position in two other species. The set of preferentially copy number regulated genes showed a reduced deviation of oriC distance in the course of evolution than promoter regulated genes. Hence, copy number regulation forces genes to keep their oriC distance. For related species with higher copy numbers, these genes are automatically expressed at higher levels during exponential phase. This could be a simple mechanism to shift up metabolism output for fast growth during adaptation to new environments (Bremer et al., 1996) and is consistent with the spontaneous emergence of fast growing bacteria in various branches of the bacterial kingdom (Couturier and Rocha, 2006). More compellingly, depending on the impact of copy numbers in these species, genes differentially expressed between exponential and stationary phase were more or less sorted along the oriC-ter axis. For the slower growing plant pathogen *D. dadantii*, genes relevant during exponential phase were less driven toward oriC than in *E. coli*, whereas in the fast growing *V. natriegens* those genes were significantly shifted toward oriC compared to *E. coli*. An extension to various clades in the bacterial kingdom revealed a connection between the growth rate of a bacterium and the positional conservation along the oriC-ter axis. The higher the growth rate of an organism, the more pronounced the sorting along the oriC-ter axis. Hence, the copy number effect is more exploited in fast growing bacteria. Couturier and Rocha speculated on the role of DNA copy numbers in shaping the bacterial chromosome during evolution (Couturier and Rocha, 2006). They observed a tendency for highly expressed genes to be placed near oriC. The growth rate dependent gene migration observed in the three investigated species supports this observation. Furthermore, the detected symmetry of the replichores supports their idea that operons relevant for a pathway are either situated nearby or at least symmetrically on both replichores. The symmetric placement avoids regulatory biases of parts of a pathway introduced by diverging copy number variations under different growth conditions. The observation in both gram-positive and gram-negative bacteria indicates a fundamental evolutionary concept of gene regulation and chromosome architecture coupled to replication dynamics and growth rates.

Our findings may have various consequences. Copy numbers impact expression patterns of exponentially growing cells. The analysis of regulatory relationships using mutants that often exhibit growth defects may be biased. A reduced copy number effect of the slow growing mutant compared to the wild type may systematically alter the set of differentially expressed genes and thereby indicate false regulatory interactions. Also, other copy number modifications including over initiation of replication or replication stalling can be detected by copy number analysis and can be corrected. We have shown that copy number effects can be computationally removed with the help of copy number analysis by DNA-sequencing (see Supplementary Figure S4) to be consistent with biological reality. This can be coupled with the verification of mutants by DNA sequencing and would therefore not lead to extra expenses. From the point of evolution, copy numbers are an interesting regulatory concept. Gene expression can be changed smoothly by shifting the gene along the oriC-ter axis with a range of several folds without the expense of regulatory proteins. As copy number regulation is fundamentally different from promoter regulation both can be modified independently with minimal crosstalk, which allows for fast evolutionary optimization processes. For the emerging field of synthetic biology, the copy number effect can be exploited by strategic positioning of metabolic pathways minimizing regulatory complexity. Especially for biotechnological applications with fast growing organisms, the continuous copy number gradient between oriC and ter is an ideal tuning vehicle for pathway integration (Slager and Veening, 2016; Scholz et al., 2019; Yubero and Poyatos, 2020; Visser et al., 2022).

## Materials and methods

### Strain cultivation and sequencing

The INV and WT strains were cultivated in LB medium (10g/L tryptone, 5g/L yeast extract, 10g/L NaCl) at 37°C under aerobic conditions in flasks with shaking at 200 rpm. For the analyzes of the exponential phase, both strains were harvested at an OD_600_ of 0.3 pelleted and immediately suspended in RNA *later* (Thermo Fisher) according to the manufacturer's instructions. The stationary phases of the WT and INV strains were harvested when no significant changes in OD_600_ could be observed for about 20 minutes. Subsequently, cells were also pelleted and suspended in RNA*later*. Samples were then split for DNA- and RNA-sequencing. Isolation of bacterial genomic DNA was performed according to Bruhn et al. (2018). For RNA-sequencing, lysis of cells and subsequent isolation of total RNA were carried out using the lysing matrix B/FastPrep sample preparation system (MP Biomedicals) and the miRNeasy Mini Kit (Qiagen), respectively. Ribosomal RNA depletion (RNA) and library preparation (RNA/DNA) was conducted by Eurofins Genomics using the Illumina Technology (strand-specific; paired-end; 2x150bp read length). All samples (INV/WT exponential and stationary phase) were carried out in biological triplicates. For the comparative genomics analysis of three species (*Dickeya dadantii* 3937; *Escherichia coli* MG1655; *Vibrio natriegens* ATCC 14048), all species were grown in rich medium. For *D. dadantii* and *E. coli* LB medium was used. For the halophilic *V. natriegens* LBV2 (LB + 204mM NaCl, 4.2mM KCl, 20.14mM MgCl_2_) was used. All three species were grown under aerobic conditions in baffled flasks with orbital shaking at 200 rpm. For optimal growth, *E. coli* and *V. natriegens* were grown at 37°C and *D. dadantii* at 30vC. Cells were harvested at OD_600_=0.3 in mid-exponential phase. DNA was also extracted according to Bruhn et al. (2018) and Illumina-sequenced by Eurofins Genomics yielding 5M 150bp paired-end reads.

### Regulatory and functional data

Data concerning sigma factor and transcription factor regulation was obtained from regulonDB (v10.9). For chromosomal sigma factor distribution, genes solely regulated by the respective sigma factor were selected. Data concerning functional identity were derived from the NCBI COG database (https://www.ncbi.nlm.nih.gov/research/cog-project/).

### Construction of the inversion (INV) and reference strain (WT)

For the construction of the inversion strain (INV) to dissect the impact of promoter regulation and copy number effect as well as for reference strain (WT) we used CRISPR SWAPnDROP to make all relevant chromosomal changes in *E. coli* MG1655 (Teufel et al., 2022). For each deletion and insertion, a different (pSwap) plasmid was constructed harboring homology regions, sgRNAs and inserts. All primers used for the amplification of the homology regions, for the sgRNA construction as well as for each insert are available in the Supplementary material. An overview of each chromosomal edit done in each of the strains is given in Table 1.

**Table 1:** Chromosomal edits of the *E. coli* INV and WT strain.

**Edit**	**Strain**	**Purpose**
Δtus	INV/WT	Ter-site inactivation
oriC::oriSFRT	INV/WT	New oriS with flanking FRT sites for removal
insPQ::oriC	INV/WT	Relocation of oriC
FRT insertion downstream of rrnE opeorn	INV/WT	Inversion of the rrnCABE operons
FRTm insertion upstream of rrnH operon	INV/WT	Inversion of the rrnH operon
FRTm insertion downstream of rrnH operon	INV/WT	Inversion of the rrnH operon
loxP511 insertion upstream of rrnG operon	INV/WT	Inversion of the rrnG operon
loxP511 insertion downstream of rrnG operon	INV/WT	Inversion of the rrnG operon
loxP insertion upstream of rrnD operon	INV/WT	Inversion of the rrnD operon
loxP insertion downstream of rrnD operon	INV/WT	Inversion of the rrnD operon
ΔoriC(insPQ)	WT	Reconstitution of the wild type
oriSFRT::oriC	WT	Reconstitution of the wild type

### Analysis of copy numbers and marker frequency

DNA read mapping was done with the R QuasR package. Marker frequency analysis (MFA) was performed to measure copy number (Maduike et al., 2014; Ivanova et al., 2015; Kemter et al., 2018). Genome coverage of exponential phase samples was first averaged over 5kb sliding windows relative to the corresponding stationary phase to get robust estimates of local copy numbers (see data points in MFA plots). A log_2_ linear regression of local copy numbers was performed for each replichore separately. The intersection ordinate of the two replichore regression curves was used as oriC and terminus (ter) copy number estimates. The data were normalized to a terminus copy number of 1 to simplify illustrations. For copy number difference, the fold change between the regression curves of the investigated growth phases and strains was calculated at the corresponding locus. For copy numbers of individual genes, the ordinate of the respective replichore regression curve at the gene locus was used.

### Expression analyzes

RNA-sequencing reads of each gene were first normalized for gene length and the total number of reads in each sample. All samples of this study were quantile-normalized in one batch to harmonize differences in overall gene expression distributions caused by technical variation. For the expression bias analyzes, the differences in the number of up- and down-regulated genes between the growth phases were determined for sliding windows of 300 genes. The chromosomal location of each window was set to the average location of all genes in the window. For the fold change analyzes, the average gene expression fold changes between the growth phases were determined for sliding windows of 300 genes. As the spatial expression pattern represents a systematic bias in expression data, the average fold changes were further corrected for relative frequency biases. This systemic bias originates from the imbalance of relative frequency when one component is enriched leading to a depletion of all other components. In the concrete case, copy number causes an increase of oriC-proximal gene expression levels, which in turn reduces expression levels of the terminus-proximal genes. This results in negative average fold change values in the terminus region. However, we assume the average fold change to be 1 at the chromosomal location where no copy number difference is present between samples. Therefore, we corrected the fold changes accordingly. The location of no copy number difference was first extracted from the copy number difference curve. The average fold change bias at that location was determined by taking the ordinate value of a regression curve of the fold change data on both replichores. All fold changes were corrected for that ordinate value. In the spatial analyzes of WT and INV, reference chromosome coordinates were set to wild type. The inversion in the INV strain causes new neighborhoods of genes at the break points of the inversion. Therefore, windows spanning these breakpoints contain gene sets that are not present in WT (e.g., WT oriC-proximal genes paired with terminus-proximal genes). These windows were omitted in the analysis as no counterpart was present in WT.

### Analysis of gene migration in *Dickeya dadantii, Escherichia coli*, and *Vibrio natriegens*

Orthologs of genes in the three species were determined using proteinortho v6 (Lechner et al., 2011). Only orthologs with a single copy (no paralogs) in all three species were considered. Gene positions were transformed to relative oriC proximities and normalized by half of the chromosome size. Consequently, values range between 1 (oriC) and 0 (ter).

For the analysis of variability of gene position, promoter and copy number regulated genes were split into 3 subsets with increasing stringency of the regulatory type (promoter or copy number). For each gene in a set the difference of oriC proximity was determined for *E. coli* vs *D. dadantii* and *E. coli* vs *V. natriegens*. The two resulting differences were averaged to reduce species-specific biases. Then, the average and standard errors of these averaged differences were determined for the different sets.

For the comparison of ortholog positions in the three species, genes significantly up-regulated (*p*-value <0.05) in exponential phase relative to stationary phase of *E. coli* WT expression data were chosen.

### Significance of functional groups

The significance of functional groups was determined by generating 1,000 random sets of genes of the same size as the set of predominantly copy number regulated genes. For these sets mean frequencies **m** and the corresponding standard deviations **s** for functional groups were determined to compute a z-score **z**


(2) $$\begin{array}{l} z\left(x\right)=\frac{x-m}{s} \end{array}$$

for each functional group, where x is the number of genes in the respective function group of the experimental data.

### Comparative genomics analysis of bacterial chromosome arrangement

The full NCBI set of completely assembled genomes was first screened for NCBI taxonomy information to cluster species according to phylogenetic categories. The remaining set was split into distinct phylogenetic classes that were analyzed separately. To cover the diversity of a class and avoid a representation bias, one species was selected out of every family of the class. Each species was a randomly selected representative species of its family, according to the NCBI database. The family category was chosen to select species with a defined range of evolutionary distance for later comparison. Furthermore, categories below a family with closer evolutionary distance yielded little chromosomal diversity for analysis. Species within a class were split into the set of fast or slow growing species. This was done by the number of 16S rRNAs that correlate with growth speed. The number of 16S rRNA was extracted from the species annotation files (GFF3) provided by NCBI. Data about the doubling times were taken from Couturier and Rocha (2006). For all species pairs within a set, orthologs were determined using proteinortho v6 (Lechner et al., 2011). Only orthologs with a single copy (no paralogs) in the two species of a pair were considered to determine reliable chromosome positions of orthologs. The oriC-ter axis was determined by finding the putative oriC position in both species that give rise to the best Pearson correlation coefficient of distances to oriC (see Section Constellation analysis). These correlation coefficients were used as an indicator of the strength of oriC-ter axis symmetry.

### Constellation analysis

For most species, oriC position is not determined. However, the oriC position causes a chromosomal symmetry due to positional conservation of genes relative to oriC (Sobetzko et al., 2012). This can be used to determine the oriC-ter axis. To determine the axis, the best overlay of chromosomes of two related species is determined. First, an arbitrary oriC position is assigned individually to both chromosomes (e.g., position 100,000 for species 1 and position 500,000 for species 2). The relative distances to all genes on both chromosomes to the assigned oriCs are determined (see Figure 5A top row). For all orthologous gene pairs of the two species, the distance to the respective oriC is compared yielding a correlation coefficient (Pearson correlation coefficient). This approach is repeated with other arbitrary oriC positions until all combinations were tested (see Figure 5B). The positions yielding the highest correlation are taken to be oriC. In (see Figure 5B) several optima are present due to intrinsic symmetries. For example, taking the correct oriC position of *E. coli* and *D. datantii* yields the same maximal correlation as taking the correct terminus position of *E. coli* and the terminus position of *D. datantii* as this directly infers that oriC is also superimposed. Which of the two optima are chosen has no impact on further analysis in this study as the level of the correlation is central not the related positions that yielded the maximal correlation. To distinguish which optimum is associated with oriC and ter, additional information such as *dnaA* or ribosomal operon location can be used. However, this is not relevant for the study. The strength of the correlation is an indicator of the conservation of gene position relative to oriC. For the analysis, it is important to consider the total evolutionary distance, as closely related species, in general, show a higher degree of conservation. This can be accomplished by choosing species within the same phylogenetic ranges (e.g., class, family, phylum etc.). Furthermore, there is a lower limit for this method, when little chromosomal rearrangements occurred between species. In this case, constellation analysis indicates this by a diagonal line of similarly high correlations. This is caused by the fact that correlation remains stable when rotating putative oriC positions on both chromosomes simultaneously (e.g., moving in 5000 bp steps in a clockwise direction). If gene positions are similar on both chromosomes, also distances to the oriCs change accordingly during synchronous rotation and retain the same correlation coefficient. The more genes have moved to other locations e.g., the opposite replichore, the more correlation differs during synchronous rotation. In Figure 5B a faint diagonal line can still be seen, indicating a small percentage of orthologous genes with a similar chromosome position in both species. As long as the two optima can still be clearly determined, the method is applicable.

## Data availability statement

The datasets presented in this study can be found in online repositories. The names of the repository/repositories and accession number(s) can be found in the article/Supplementary material.

## Author contributions

PS and WH conceived the presented project. MT and PS planned and performed the experiments. MT, WH, and PS analyzed the data. All authors discussed the results and contributed to the final manuscript.

## References

[B1] Balke V. L. Gralla J. (1987). Changes in the linking number of supercoiled dna accompany growth transitions in escherichia coli. J. Bacteriol. 169, 4499–4506. 10.1128/jb.169.10.4499-4506.1987 3308843 PMC213814

[B2] Beber M. E. Sobetzko P. Muskhelishvili G. Htt M. T. (2016). Interplay of digital and analog control in time-resolved gene expression profiles. EPJ Nonlinear Biomed. Phys. 4, 1–16. 10.1140/epjnbp/s40366-016-0035-7

[B3] Bipatnath M. Dennis P. P. Bremer H. (1998). Initiation and velocity of chromosome replication in escherichia coli b/r and k-12. J. Bacteriol. 180, 265–273. 10.1128/JB.180.2.265-273.1998 9440515 PMC106881

[B4] Blakely G. Colloms S. May G. Burke M. Sherratt D. (1991). Escherichia coli XerC recombinase is required for chromosomal segregation at cell division. New Biol. 3, 789–798. 1931824

[B5] Block D. H. Hussein R. Liang L. W. Lim H. N. (2012). Regulatory consequences of gene translocation in bacteria. Nucleic Acids Res. 40, 8979–8992. 10.1093/nar/gks694 22833608 PMC3467084

[B6] Blot N. Mavathur R. Geertz M. Travers A. Muskhelishvili G. (2006). Homeostatic regulation of supercoiling sensitivity coordinates transcription of the bacterial genome. EMBO Rep. 7, 710–715. 10.1038/sj.embor.7400729 16799466 PMC1500834

[B7] Bremer H. Dennis P. P. (1996). Escherichia coli and Salmonella: Cellular and Molecular Biology. Washington (DC): American Society for Microbiology. 1553–1569.

[B8] Bruhn M. Schindler D. Kemter F. S. Wiley M. R. Chase K. Koroleva G. I.. (2018). Functionality of two origins of replication in vibrio cholerae strains with a single chromosome. Front. Microbiol. 9, 2932. 10.3389/fmicb.2018.02932 30559732 PMC6284228

[B9] Bryant J. A. Sellars L. E. Busby S. J. Lee D. J. (2014). Chromosome position effects on gene expression in Escherichia coli K-12. Nucleic Acids Res. 42, 11383–11392. 10.1093/nar/gku828 25209233 PMC4191405

[B10] Chandler M. G. Pritchard R. H. (1975). The effect of gene concentration and relative gene dosage on gene output in Escherichia coli. Mol. Gen. Genet. 138, 127–141. 10.1007/BF02428117 1105148

[B11] Chen D. Lilley D. M. (1999). Transcription-induced hypersupercoiling of plasmid dna. J. Molec. Biol. 285, 443–448. 10.1006/jmbi.1998.2358 9878418

[B12] Condon C. Liveris D. Squires C. Schwartz I. Squires C. L. (1995). rRNA operon multiplicity in escherichia coli and the physiological implications of rrn inactivation. J. Bacteriol. 177, 4152–4156. 10.1128/jb.177.14.4152-4156.1995 7608093 PMC177152

[B13] Couturier E. Rocha E. P. (2006). Replication-associated gene dosage effects shape the genomes of fast-growing bacteria but only for transcription and translation genes. Mol. Microbiol. 59, 1506–1518. 10.1111/j.1365-2958.2006.05046.x 16468991

[B14] de Matos Simoes R. Dehmer M. Emmert-Streib F. (2013). Interfacing cellular networks of S. cerevisiae and E. coli: connecting dynamic and genetic information. BMC Genom. 14, 324. 10.1186/1471-2164-14-324 23663484 PMC3698017

[B15] Donachie W. D. (1968). Relationship between cell size and time of initiation of DNA replication. Nature 219, 1077–1079. 10.1038/2191077a0 4876941

[B16] Egan E. S. Løbner-Olesen A. Waldor M. K. (2004). Synchronous replication initiation of the two vibrio cholerae chromosomes. Curr. Biol. 14, R501–R502. 10.1016/j.cub.2004.06.036 15242627

[B17] El Houdaigui B. Forquet R. Hindr T. Schneider D. Nasser W. Reverchon S.. (2019). Bacterial genome architecture shapes global transcriptional regulation by DNA supercoiling. Nucleic Acids Res. 47, 5648–5657. 10.1093/nar/gkz300 31216038 PMC6582348

[B18] el-Hajj H. H. Zhang H. Weiss B. (1988). Lethality of a dut (deoxyuridine triphosphatase) mutation in Escherichia coli. J. Bacteriol. 170, 1069–1075. 10.1128/jb.170.3.1069-1075.1988 2830228 PMC210875

[B19] Ferullo D. J. Cooper D. L. Moore H. R. Lovett S. T. (2009). Cell cycle synchronization of Escherichia coli using the stringent response, with fluorescence labeling assays for DNA content and replication. Methods 48, 8–13. 10.1016/j.ymeth.2009.02.010 19245839 PMC2746677

[B20] Freddolino P. L. Amemiya H. M. Goss T. J. Tavazoie S. (2021). Dynamic landscape of protein occupancy across the Escherichia coli chromosome. PLoS Biol. 19, e3001306. 10.1371/journal.pbio.3001306 34170902 PMC8282354

[B21] Gao F. Zhang C.-T. (2007). DoriC: a database of oriC regions in bacterial genomes. Bioinformatics 23, 1866–1867. 10.1093/bioinformatics/btm255 17496319

[B22] Hartl J. Kiefer P. Meyer F. Vorholt J. A. (2017). Longevity of major coenzymes allows minimal de novo synthesis in microorganisms. Nat. Microbiol. 2, 17073. 10.1038/nmicrobiol.2017.73 28504670 PMC6241834

[B23] Hill T. M. (1992). Arrest of bacterial DNA replication. Annu. Rev. Microbiol. 46, 603–633. 10.1146/annurev.mi.46.100192.003131 1444268

[B24] Huang Y.-P. Ito J. (1999). Dna polymerase c of the thermophilic bacterium thermus aquaticus: classification and phylogenetic analysis of the family c dna polymerases. J. Molec. Evol. 48, 756–769. 10.1007/PL00006520 10229580

[B25] Ivanova D. Taylor T. Smith S. L. Dimude J. U. Upton A. L. Mehrjouy M. M.. (2015). Shaping the landscape of the Escherichia coli chromosome: replication-transcription encounters in cells with an ectopic replication origin. Nucleic Acids Res. 43, 7865–7877. 10.1093/nar/gkv704 26160884 PMC4652752

[B26] Jinks-Robertson S. Nomura M. (1987). Ribosomes and tRNA. Escherichia coli Salmonella Typhimur Molec. Cell Biol. 2, 1358–1385.

[B27] Jishage M. Kvint K. Shingler V. m T. (2002). Regulation of sigma factor competition by the alarmone ppGpp. Genes Dev. 16, 1260–1270. 10.1101/gad.227902 12023304 PMC186289

[B28] Kemter F. S. Messerschmidt S. J. Schallopp N. Sobetzko P. Lang E. Bunk B.. (2018). Synchronous termination of replication of the two chromosomes is an evolutionary selected feature in Vibrionaceae. PLoS Genet. 14, e1007251. 10.1371/journal.pgen.1007251 29505558 PMC5854411

[B29] Klein C. A. Teufel M. Weile C. J. Sobetzko P. (2021). The bacterial promoter spacer modulates promoter strength and timing by length, TG-motifs and DNA supercoiling sensitivity. Sci. Rep. 11, 24399. 10.1038/s41598-021-03817-4 34937877 PMC8695583

[B30] Kosmidis K. Jablonski K. P. Muskhelishvili G. tt M. T. (2020). Chromosomal origin of replication coordinates logically distinct types of bacterial genetic regulation. NPJ Syst. Biol. Appl. 6, 5. 10.1038/s41540-020-0124-1 32066730 PMC7026169

[B31] Lal A. Dhar A. Trostel A. Kouzine F. Seshasayee A. S. Adhya S. (2016). Genome scale patterns of supercoiling in a bacterial chromosome. Nat. Commun. 7, 11055. 10.1038/ncomms11055 27025941 PMC4820846

[B32] Lechner M. Findeiss S. Steiner L. Marz M. Stadler P. F. Prohaska S. J. (2011). Proteinortho: detection of (co-)orthologs in large-scale analysis. BMC Bioinform. 12, 124. 10.1186/1471-2105-12-124 21526987 PMC3114741

[B33] Liu L. F. Wang J. C. (1987). Supercoiling of the dna template during transcription. Proc. Nat. Acad. Sci. 84, 7024–7027. 10.1073/pnas.84.20.7024 2823250 PMC299221

[B34] Lu P. (1997). The lac operon: A short history of a genetic paradigm. Science 275, 938–939. 10.1126/science.275.5302.938

[B35] Maduike N. Z. Tehranchi A. K. Wang J. D. Kreuzer K. N. (2014). Replication of the Escherichia coli chromosome in RNase HI-deficient cells: multiple initiation regions and fork dynamics. Mol. Microbiol. 91, 39–56. 10.1111/mmi.12440 24164596 PMC3926323

[B36] Marr C. Geertz M. Htt M. T. Muskhelishvili G. (2008). Dissecting the logical types of network control in gene expression profiles. BMC Syst. Biol. 2, 18. 10.1186/1752-0509-2-18 18284674 PMC2263018

[B37] Martìnez-Antonio A. Janga S. C. Thieffry D. (2008). Functional organisation of Escherichia coli transcriptional regulatory network. J. Mol. Biol. 381, 238–247. 10.1016/j.jmb.2008.05.054 18599074 PMC2726282

[B38] Martis B S. Forquet R. Reverchon S. Nasser W. Meyer S. (2019). DNA supercoiling: an ancestral regulator of gene expression in pathogenic bacteria? Comput. Struct. Biotechnol. J. 17, 1047–1055. 10.1016/j.csbj.2019.07.013 31452857 PMC6700405

[B39] McGovern V. Higgins N. P. Chiz R. S. Jaworski A. (1994). H-NS over-expression induces an artificial stationary phase by silencing global transcription. Biochimie 76, 1019–1029. 10.1016/0300-9084(94)90026-4 7748923

[B40] Menzel R. Gellert M. (1983). Regulation of the genes for e. coli dna gyrase: homeostatic control of dna supercoiling. Cell 34, 105–113. 10.1016/0092-8674(83)90140-X 6309403

[B41] Mercier R. Petit M. A. Schbath S. Robin S. El Karoui M. Boccard F.. (2008). The MatP/matS site-specific system organizes the terminus region of the E. coli chromosome into a macrodomain. Cell 135, 475–485. 10.1016/j.cell.2008.08.031 18984159

[B42] Missirlis P. I. Smailus D. E. Holt R. A. (2006). A high-throughput screen identifying sequence and promiscuity characteristics of the loxP spacer region in Cre-mediated recombination. BMC Genomics 7, 73. 10.1186/1471-2164-7-73 16595017 PMC1479339

[B43] Pineau M. Martis B S. Forquet R. Baude J. Villard C. Grand L.. (2022). What is a supercoiling-sensitive gene? Insights from topoisomerase I inhibition in the Gram-negative bacterium Dickeya dadantii. Nucleic Acids Res. 50, 9149–9161. 10.1093/nar/gkac679 35950487 PMC9458453

[B44] Riebet E. Raibaud O. (1991). Supercoiling is essential for the formation and stability of the initiation complex at the divergent malep and malkp promoters. J. Molec. Biol. 218, 529–542. 10.1016/0022-2836(91)90699-7 2016744

[B45] Schmid M. B. Roth J. R. (1987). Gene location affects expression level in Salmonella typhimurium. J. Bacteriol. 169, 2872–2875. 10.1128/jb.169.6.2872-2875.1987 3294809 PMC212203

[B46] Scholz S. A. Diao R. Wolfe M. B. Fivenson E. M. Lin X. N. Freddolino P. L. (2019). High-resolution mapping of the escherichia coli chromosome reveals positions of high and low transcription. Cell Syst. 8, 212–225. 10.1016/j.cels.2019.02.004 30904377 PMC6508686

[B47] Schultz J. E. Latter G. I. Matin A. (1988). Differential regulation by cyclic AMP of starvation protein synthesis in Escherichia coli. J. Bacteriol. 170, 3903–3909. 10.1128/jb.170.9.3903-3909.1988 2842291 PMC211388

[B48] Skovgaard O. Bak M. bner Olesen A. Tommerup N. (2011). Genome-wide detection of chromosomal rearrangements, indels, and mutations in circular chromosomes by short read sequencing. Genome Res. 21, 1388–1393. 10.1101/gr.117416.110 21555365 PMC3149504

[B49] Slack A. Thornton P. C. Magner D. B. Rosenberg S. M. Hastings P. J. (2006). On the mechanism of gene amplification induced under stress in *Escherichia coli. PLoS Genet*. 2, e48. 10.1371/journal.pgen.0020048 16604155 PMC1428787

[B50] Slager J. Veening J. W. (2016). Hard-wired control of bacterial processes by chromosomal gene location. Trends Microbiol. 24, 788–800. 10.1016/j.tim.2016.06.003 27364121 PMC5034851

[B51] Sobetzko P. (2016). Transcription-coupled DNA supercoiling dictates the chromosomal arrangement of bacterial genes. Nucleic Acids Res. 44, 1514–1524. 10.1093/nar/gkw007 26783203 PMC4770239

[B52] Sobetzko P. Glinkowska M. Travers A. Muskhelishvili G. (2013). DNA thermodynamic stability and supercoil dynamics determine the gene expression program during the bacterial growth cycle. Mol. Biosyst. 9, 1643–1651. 10.1039/c3mb25515h 23493878

[B53] Sobetzko P. Travers A. Muskhelishvili G. (2012). Gene order and chromosome dynamics coordinate spatiotemporal gene expression during the bacterial growth cycle. Proc. Natl. Acad. Sci. USA. 109, 42–50. 22184251 10.1073/pnas.1108229109 PMC3258614

[B54] Sousa C. de Lorenzo V. Cebolla A. (1997). Modulation of gene expression through chromosomal positioning in Escherichia coli. Microbiology 143, 2071–2078. 10.1099/00221287-143-6-2071 9202482

[B55] Stülke J. Hillen W. (1999). Carbon catabolite repression in bacteria. Curr. Opin. Microbiol. 2, 195–201. 10.1016/S1369-5274(99)80034-4 10322165

[B56] Tani T. H. Khodursky A. Blumenthal R. M. Brown P. O. Matthews R. G. (2002). Adaptation to famine: a family of stationary-phase genes revealed by microarray analysis. Proc. Natl. Acad. Sci. U S A 99, 13471–13476. 10.1073/pnas.212510999 12374860 PMC129697

[B57] Teufel M. Klein C. A. Mager M. Sobetzko P. (2022). A multifunctional system for genome editing and large-scale interspecies gene transfer. Nat. Commun. 13, 3430. 10.1038/s41467-022-30843-1 35701417 PMC9198041

[B58] Turan S. Kuehle J. Schambach A. Baum C. Bode J. (2010). Multiplexing RMCE: versatile extensions of the Flp-recombinase-mediated cassette-exchange technology. J. Mol. Biol. 402, 52–69. 10.1016/j.jmb.2010.07.015 20650281

[B59] Valens M. Penaud S. Rossignol M. Cornet F. Boccard F. (2004). Macrodomain organization of the Escherichia coli chromosome. EMBO J. 23, 4330–4341. 10.1038/sj.emboj.7600434 15470498 PMC524398

[B60] Valens M. Thiel A. Boccard F. (2016). The MaoP/maoS Site-Specific System Organizes the Ori Region of the *E. coli* Chromosome into a Macrodomain. PLoS Genet. 12, e1006309. 10.1371/journal.pgen.1006309 27627105 PMC5023128

[B61] Visser B. J. Sharma S. Chen P. J. McMullin A. B. Bates M. L. Bates D. (2022). Psoralen mapping reveals a bacterial genome supercoiling landscape dominated by transcription. Nucl. Acids Res. 50, 4436–4449. 10.1093/nar/gkac244 35420137 PMC9071471

[B62] Yubero P. Poyatos J. F. (2020). The impact of global transcriptional regulation on bacterial gene order. iScience 23, 101029. 10.1016/j.isci.2020.101029 32283521 PMC7155222

[B63] Zampieri M. Hrl M. Hotz F. Mller N. F. Sauer U. (2019). Regulatory mechanisms underlying coordination of amino acid and glucose catabolism in Escherichia coli. Nat. Commun. 10, 3354. 10.1038/s41467-019-11331-5 31350417 PMC6659692

