# Supplementary material for: The role of replication-induced chromosomal copy numbers in spatio-temporal gene regulation and evolutionary chromosome plasticity
Source: Front Microbiol. 2023 Apr 20;14:1119878. doi: 10.3389/fmicb.2023.1119878 (PMC10157177; doi:10.3389/fmicb.2023.1119878)
Supplement: Supplementary file 1 [file Data_Sheet1_.pdf]

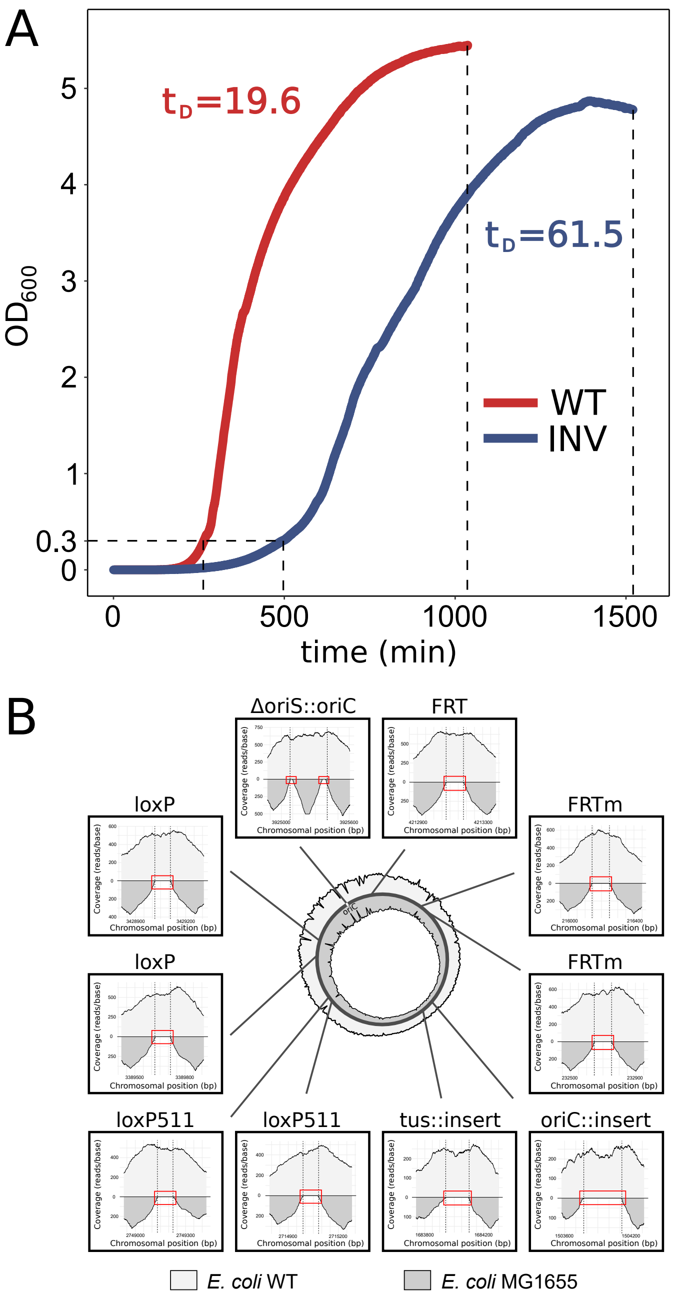

Figure S1. Growth curve and chromosomal edits in the WT and INV strains. (A) Growth curve of the WT and the INV strain in LB medium at 37°C and aeration. Doubling times (t_D) are indicated. Harvesting of exponential phase and stationary phase samples is indicated by dashed lines. **(B)** Next-Generation Sequencing of the *E. coli* WT strain. Shown is the next-generation sequencing (NGS) coverage of the *E. coli* WT strain after 12 consecutive edits (light grey) compared to its precursor *E. coli* MG1655 (dark grey). For the wild type strain, several recombination sites (FRT/loxP) as well as random DNA and origins of replication were inserted into the chromosome using iterative CRISPR SWAPnDROP genome editing. *E. coli* WT strain and MG1655 NGS reads were aligned against the WT strain reference genome and the sectors of each edited site as well as the complete genome coverage (circle) are shown. Reads at all insertion locations (dashed lines) are present for the wild type strain, while no reads are present for MG1655 (red rectangle).

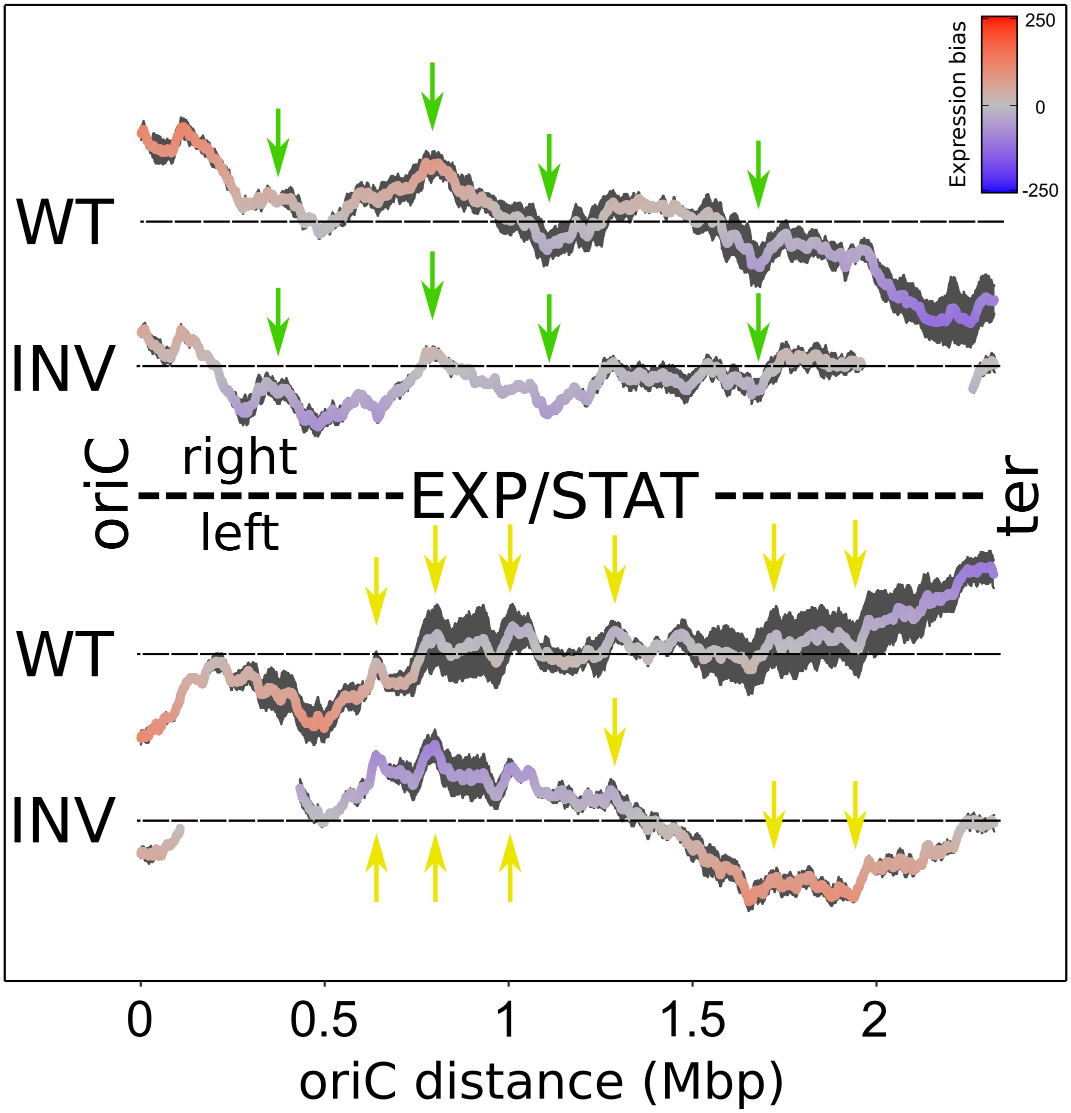

Figure S2. Comparison of the local spatial expression pattern of WT and INV strains between exponential and stationary phase. Replichores of both strains were aligned by position but shifted vertically to avoid overlapping. Expression biases of both strains are mapped against the WT chromosomal location. The zero level of each curve is indicated by a black horizontal line. Green and yellow arrows indicate characteristic local peaks on the left and the right replichore, respectively. Gaps for the INV strain are due to the absence of corresponding wild type windows comprising the inversion break points.

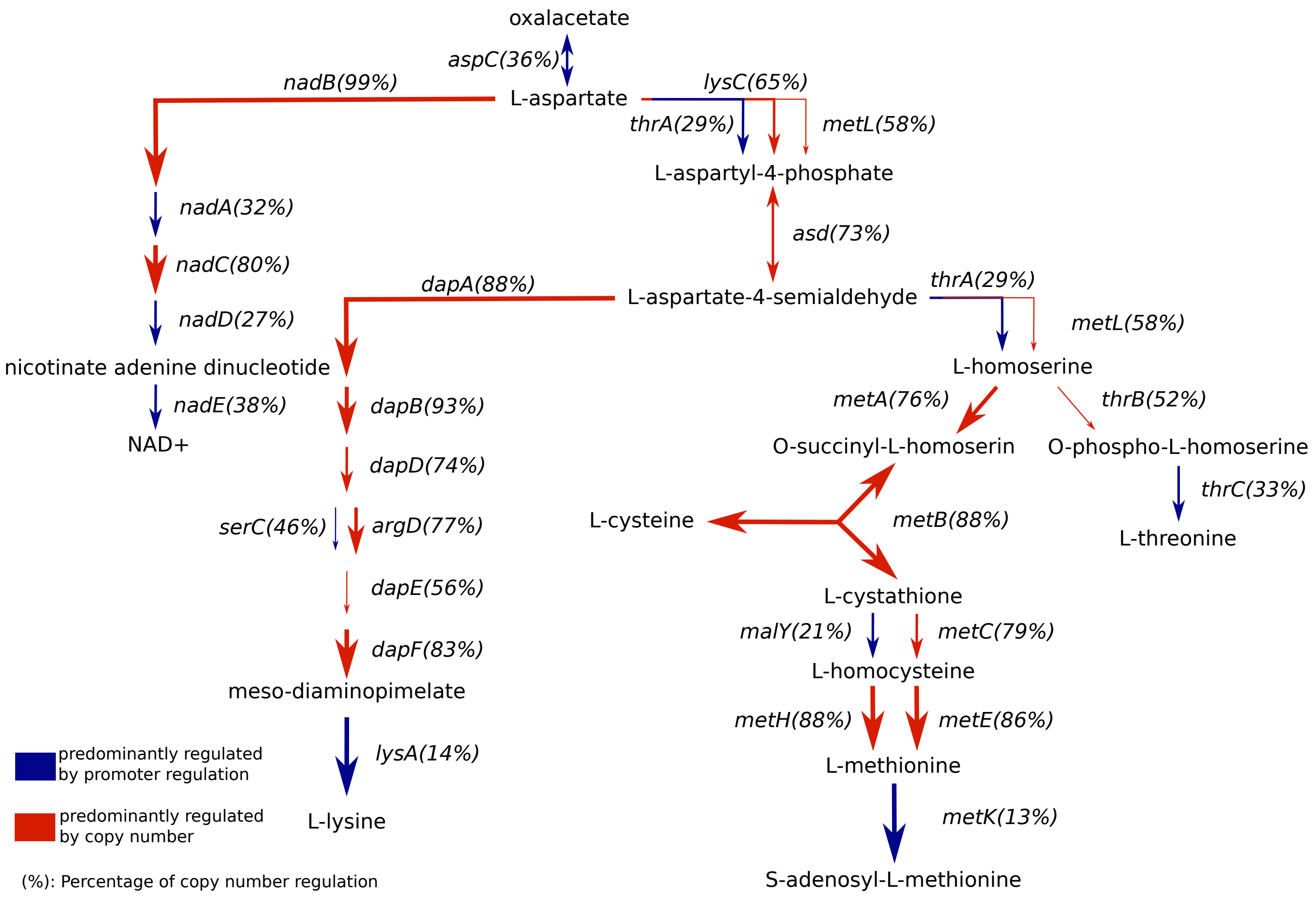

Figure S3. Impact of copy number on the aspartate pathway regulation. Shown is the aspartate pathway of *E. coli*. Red and blue arrows indicate dominant copy number and promoter regulation, respectively. The thickness of the arrow indicate the degree of the dominance also indicated in percent next to the gene coding for the enzyme involved in the enzymatic step.

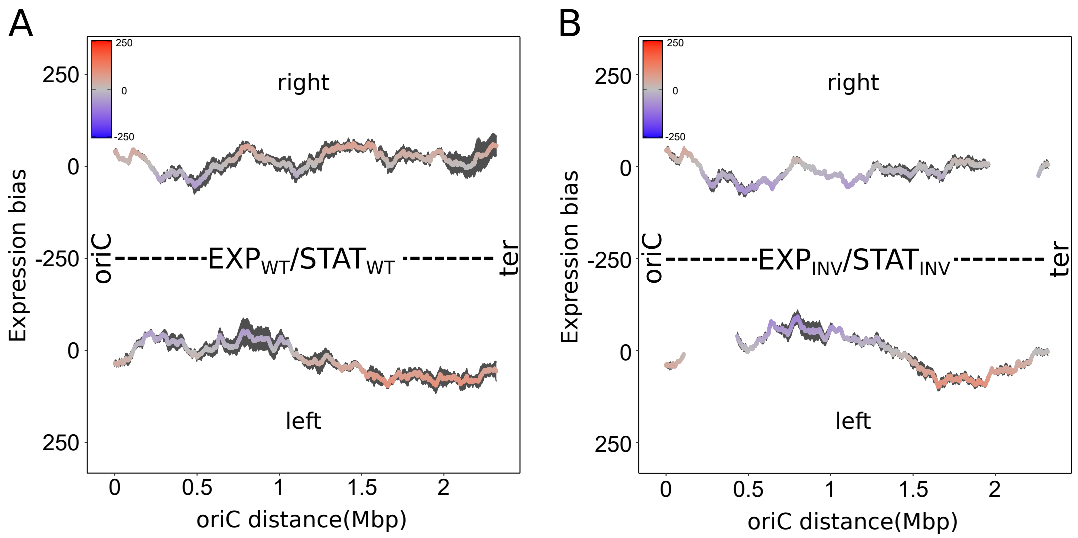

Figure S4. Computational copy number normalisation vs. biological copy number reduction. Comparison of the spatial expression pattern of WT with copy number normalisation (A) and the spatial pattern of the INV strain with a strongly reduced copy number between exponential and stationary phase (B). Colors indicate the extent of the spatial expression bias. Gaps for the INV strain are due to the absence of corresponding WT windows comprising the inversion break points.

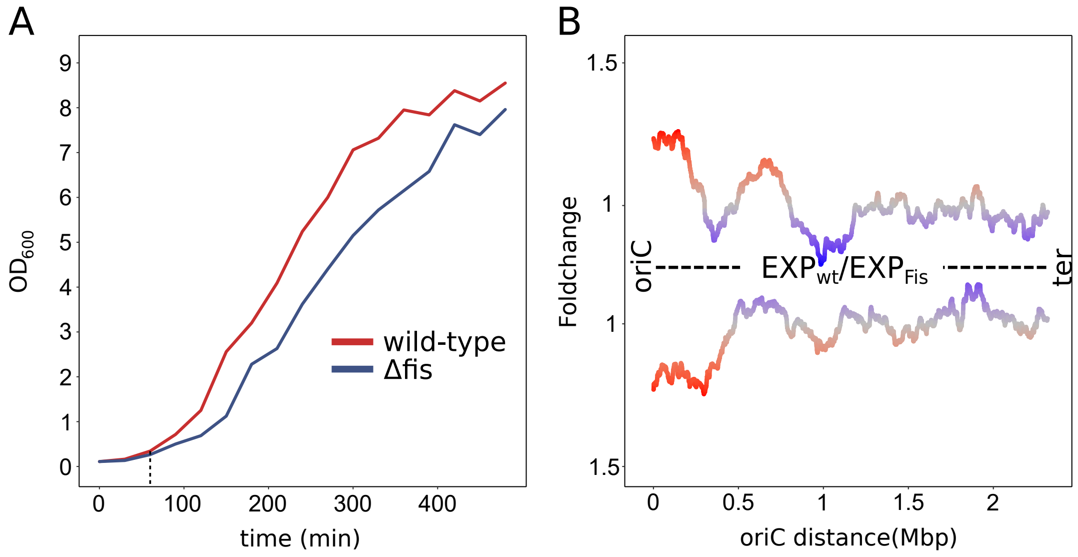

Figure S5. Putative impact of growth defects on spatial expression in mutant analysis. Expression data and growth curves were taken from Beber et al. 2016[?]. **(A)** Growth curves of *E. coli* wild type and its Fis deletion mutant. Harvesting of exponential phase samples is indicated by dashed lines. **(B)** Spatial expression pattern of *E. coli* wild type (CSH50) compared to its Fis deletion mutant. Average fold changes (wt/Δfis) of gene expression within a sliding window of 300 genes is depicted.

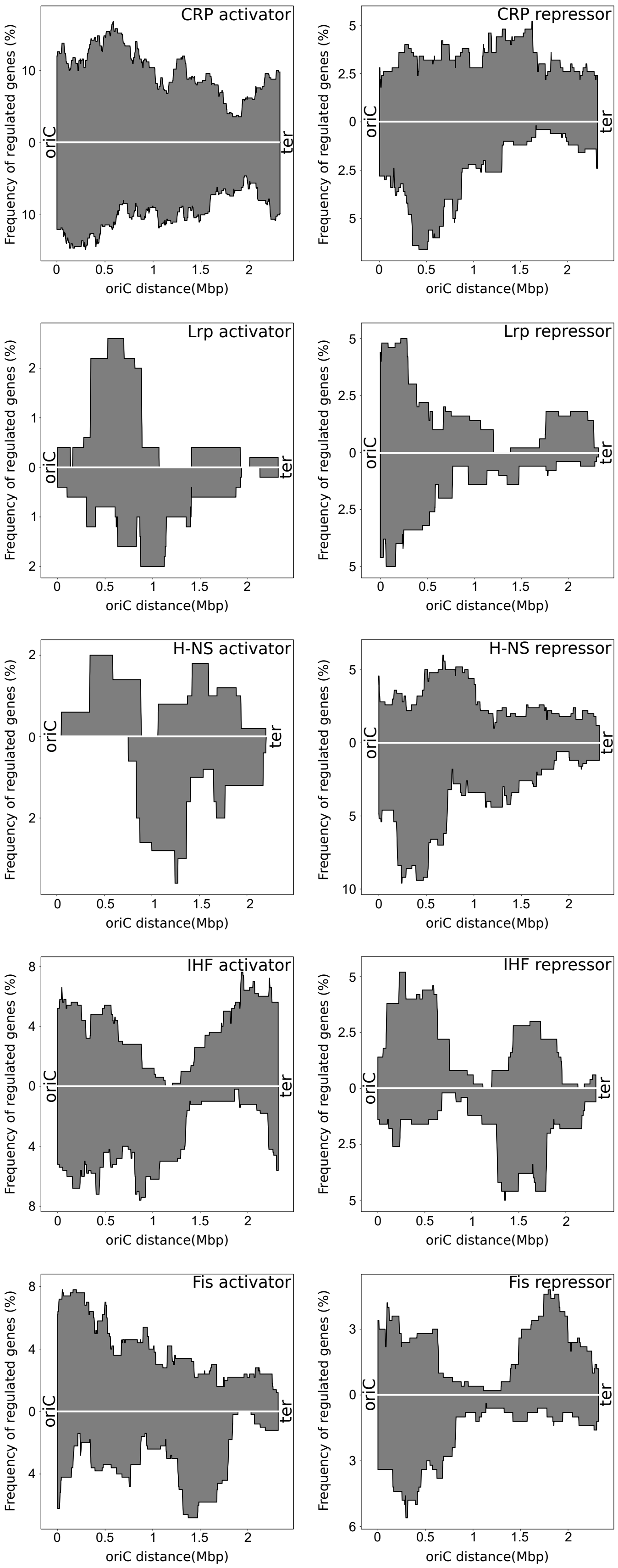

Figure S6. **Spatial frequency of genes regulated by global regulators.** Shown is the spatial frequency of genes regulated by the global regulators CRP, Lrp, H-NS, IHF and Fis subdivided according to their activation or repression activity.

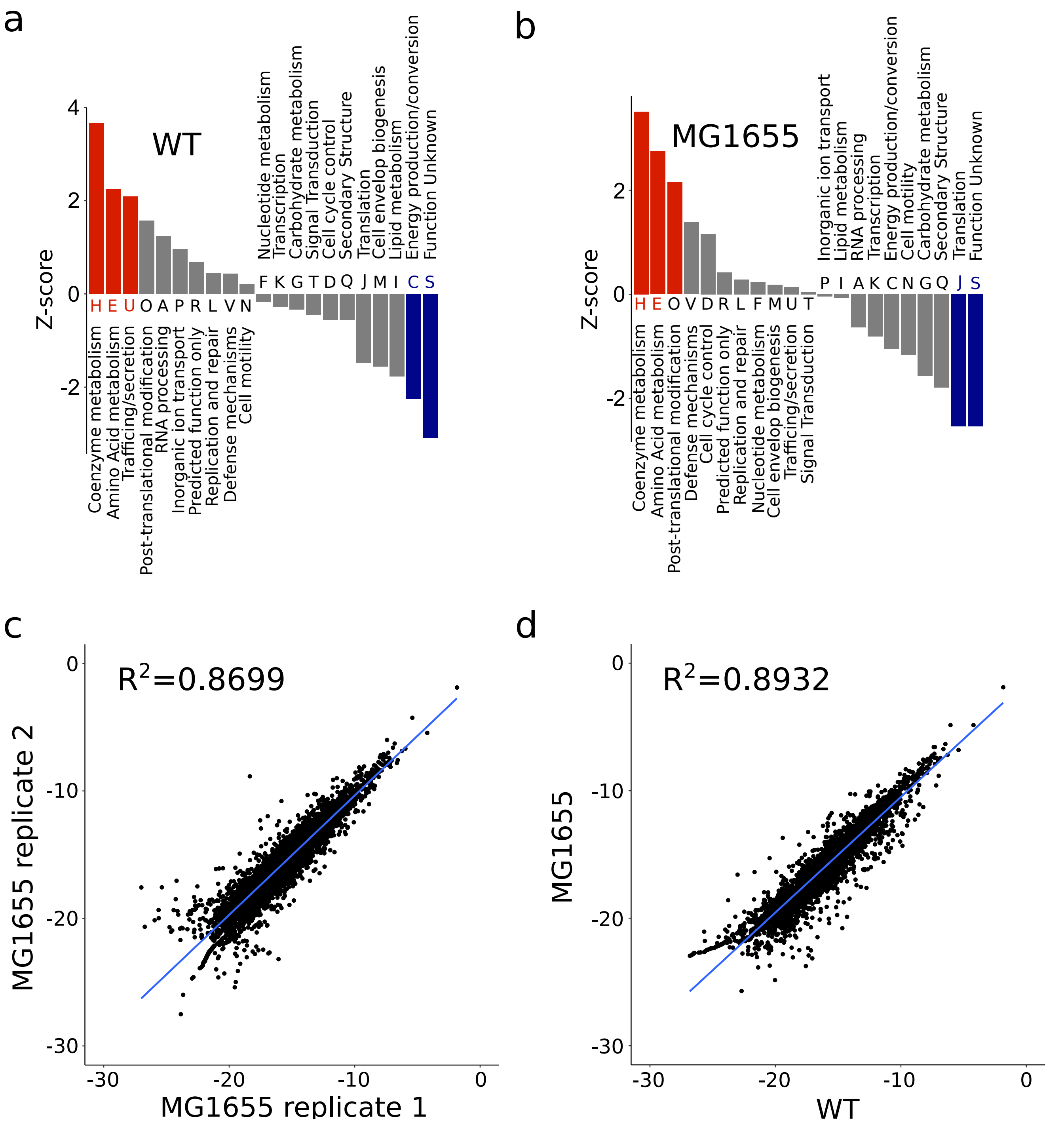

Figure S7. Comparison of the *E. coli* WT and MG1655 strains. Comparison of the functionality group analysis of the WT (a) and MG1655 (b) strain. (c) Expression data ($\log_{10}$) from MG1655 replicate 1 was plotted against MG1655 replicate 2. (d) Averaged expression data ($\log_{10}$) from MG1655 was plotted against averaged expression data from WT strain.

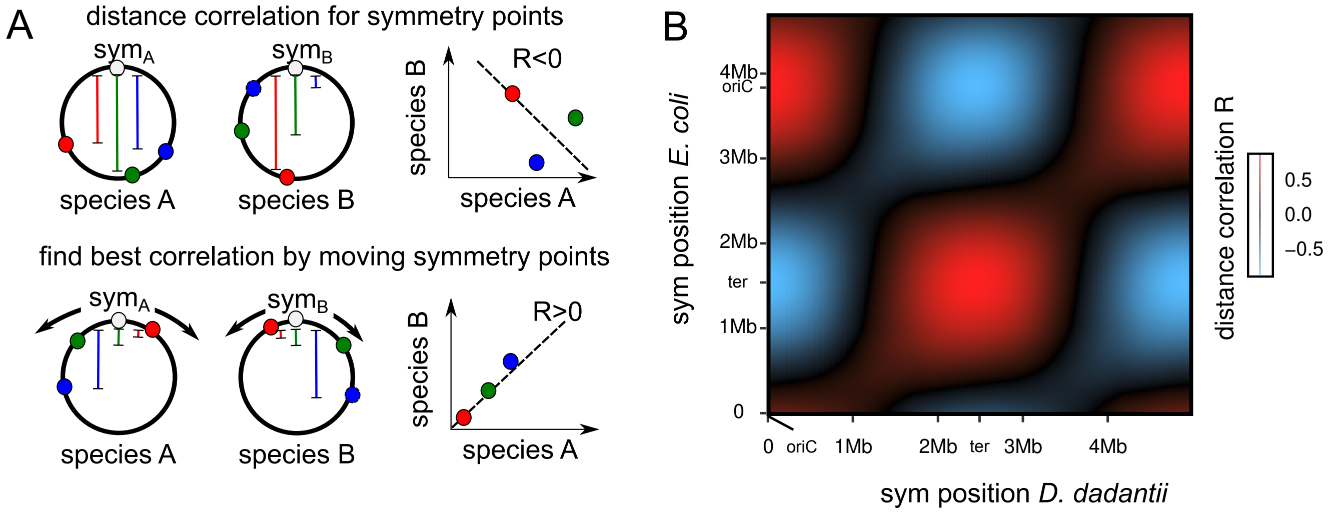

Figure S8. **(A)** Scheme of screening for the oriC-ter axis in species without known oriC. The axes in both species move through the putative oriC positions that yields the best oriC distance correlation of orthologs. **(B)** oriC-ter axis analysis for *E. coli* and *D. dadantii*. The correct oriC positions are indicated matching the maximum correlation (red).
